# Analysis of cardiovascular dynamics in pulmonary hypertensive C57BL6/J mice

**DOI:** 10.3389/fphys.2013.00355

**Published:** 2013-12-11

**Authors:** Shivendra G. Tewari, Scott M. Bugenhagen, Zhijie Wang, David A. Schreier, Brian E. Carlson, Naomi C. Chesler, Daniel A. Beard

**Affiliations:** ^1^Biotechnology and Bioengineering Center, Medical College of WisconsinMilwaukee, WI, USA; ^2^Department of Physiology, Medical College of WisconsinMilwaukee, WI, USA; ^3^Department of Biomedical Engineering, University of Wisconsin–MadisonMadison, WI, USA

**Keywords:** pulmonary hypertension, cardiac mechanics, myofiber, stress, strain, re-modeling

## Abstract

A computer model was used to analyze data on cardiac and vascular mechanics from C57BL6/J mice exposed to 0 (*n* = 4), 14 (*n* = 6), 21 (*n* = 8) and 28 (*n* = 7) days of chronic hypoxia and treatment with the VEGF receptor inhibitor SUGEN (HySu) to induce pulmonary hypertension. Data on right ventricular pressure and volume, and systemic arterial pressure obtained before, during, and after inferior vena cava occlusion were analyzed using a mathematical model of realistic ventricular mechanics coupled with a simple model of the pulmonary and systemic vascular systems. The model invokes a total of 26 adjustable parameters, which were estimated based on least-squares fitting of the data. Of the 26 adjustable parameters, 14 were set to globally constant values for the entire data set. It was necessary to adjust the remaining 12 parameters to match data from all experimental groups. Of these 12 individually adjusted parameters, three parameters representing pulmonary vascular resistance, pulmonary arterial elastance, and pulmonary arterial narrowing were found to significantly change in HySu-induced remodeling. Model analysis shows a monotonic change in these parameters as disease progressed, with approximately 130% increase in pulmonary resistance, 70% decrease in unstressed pulmonary arterial volume, and 110% increase in pulmonary arterial elastance in the 28-day group compared to the control group. These changes are consistent with prior experimental measurements. Furthermore, the 28-day data could be explained only after increasing the passive elastance of the right free wall compared to the value used for the other data sets, which is likely a consequence of the increased RV collagen accumulation found experimentally. These findings may indicate a compensatory remodeling followed by pathological remodeling of the right ventricle in HySu-induced pulmonary hypertension.

## Introduction

Pulmonary arterial hypertension (PAH) is a pathophysiological condition of sustained mean pulmonary arterial pressure greater than 25 mmHg at rest with a pulmonary capillary wedge pressure lower than 15 mmHg (Stenmark et al., 2009). It is a syndrome in which obstruction of pulmonary arteries increases pulmonary vascular resistance (PVR) leading to right ventricular (RV) hypertrophy, failure and death (Lumens et al., 2009a; Ciuclan et al., 2011). The historical median survival for untreated idiopathic PAH (IPAH) patient is 2.8 years (Gibbs, 2007). The etiology of PAH is not completely understood, with multiple factors involved in its pathogenesis (Stenmark et al., 2009; Ciuclan et al., 2011), the revelation of which is key to designing therapeutic strategies. Ciuclan et al. (2011) reported a novel murine model of PAH that is relevant to pathogenesis of human PAH and presents incipient right heart failure after 21 days of chronic hypoxia exposure with SUGEN treatment (HySu). However, they did not study the mechanical load response of RV comprehensively at the whole organ or sarcomere levels. Therefore, the effect of PAH progression on RV failure remains poorly understood.

A recent study by Wang et al. (2013) measured *in vivo* hemodynamics to characterize RV mechanics and cardiovascular function in mice exposed to HySu for 0, 14, 21, and 28 days. Progressive changes in RV function were found, suggesting a shift of RV remodeling from adaptation to maladaptive. However, the *in vivo* measurement was at the whole organ level and thus the mechanical load response of RV sarcomere was unclear. Moreover, remodeling in the pulmonary vascular bed such as arterial narrowing and stiffening cannot be directly measured from the RV pressure-volume relationships. To address these issues, a computer model of the heart and circulation is used to provide a quantitative description of the hemodynamic data of Wang et al. (2013).

The computer model simulates realistic ventricular interactions using the TriSeg model (Lumens et al., 2009b) while the circulation is simulated using a simple lumped parameter model adapted from Smith et al. (2004). It invokes a total of 26 adjustable parameters that are estimated based on least-squares fit to the measured data. Of these 26 parameters, it was necessary to adjust 12 parameters to match data for each individual animal from all experimental groups, while 14 were set to globally constant values for each individual animal from all experimental groups. Among these 12 individually adjusted parameters, three parameters representing pulmonary vascular resistance, pulmonary arterial elastance, and pulmonary arterial narrowing were found to significantly change in the diseased experimental groups. In addition, for the 28-day group it was necessary to adjust the passive stress-strain curve of right free wall (RW) from the baseline value used by Lumens et al. (2009b). Specifically, the transition point of this stress-strain relationship is modified which increases the slope of the stress-strain curve for the 28-day group, which may reflect increased collagen accumulation. Mechanical load response of the RW was calculated as area of the stress-strain curve, defined as the stroke work density per unit tissue volume. The model suggests a trend of compensatory increase in stroke work density for 14- and 21-day groups followed by pathological reduction for 28-day group. Together, these changes in RW work density potentially indicate the onset of RV dysfunction. However, the predicted trends in RW work density are not statistically significant for the number of animals studied.

## Materials and methods

### Experimental data

RV pressure, volume and systemic/aortic pressure data from Wang et al. (2013) are used to identify the mathematical framework, which simulates the effect of HySu on the cardiovascular system. Admittance based catheters were used to measure pressure and volume from C57BL6/J mice exposed to HySu for 14, 21, and 28 days to induce PAH. A normoxia control group was used by vehicle treatment for 21 days in room air conditions. After initial measurements of pressure and volume, the inferior vena cava (IVC) was occluded to alter the preload. The vena cava occlusion (VCO) was limited to a few seconds to minimize any response of the baroreflex. (Additionally, urethane anesthetic used in the experiments effectively suppressed the baroreflex response). Measurements of hematocrit, RW, left ventricular free wall (LW) plus septal tissue mass were obtained after euthanasia. For detailed experimental procedures, see Wang et al. (2013).

### Computational model

The TriSeg model (Lumens et al., 2009b) is used to describe the ventricular mechanics of the heart and is coupled with a simple model of the circulation (Smith et al., 2004). The TriSeg model, described in detail by Lumens et al. (2009b), represents the heart as three thick-walled spherical segments which meet at a junction margin encapsulating the left ventricle (LV) and RV cavity. It incorporates mechanical interactions between LW, RW and septum resulting in realistic LV and RV pump mechanics. The wall geometries are used to calculate representative myofiber strain for each wall, which is used to calculate myofiber stress using constitutive equations describing sarcomere mechanics. Myofiber stress and wall geometries, in turn, are used to calculate representative radial and axial tensile forces acting on the junction line. The septal geometry is adjusted so that equilibrium of tensile forces at the junction is maintained. For simulating the mouse heart, the TriSeg model was modified to account for physiological heart-rate changes by adapting mechanical activation of sarcomere on a beat-to-beat basis. The mechanical activation of sarcomeres in the TriSeg Model (Lumens et al., 2009b), which is physiologically related to intracellular calcium concentration, is governed by the three time-constants controlling calcium rise and decay times, and sarcomere contraction duration (see section 1 of the supplementary material). These time-constants were scaled by heart-rate accounting for physiological changes in sarcomere dynamics for each cardiac cycle (see section 1 of the supplementary material for details).

**Figure 1:**
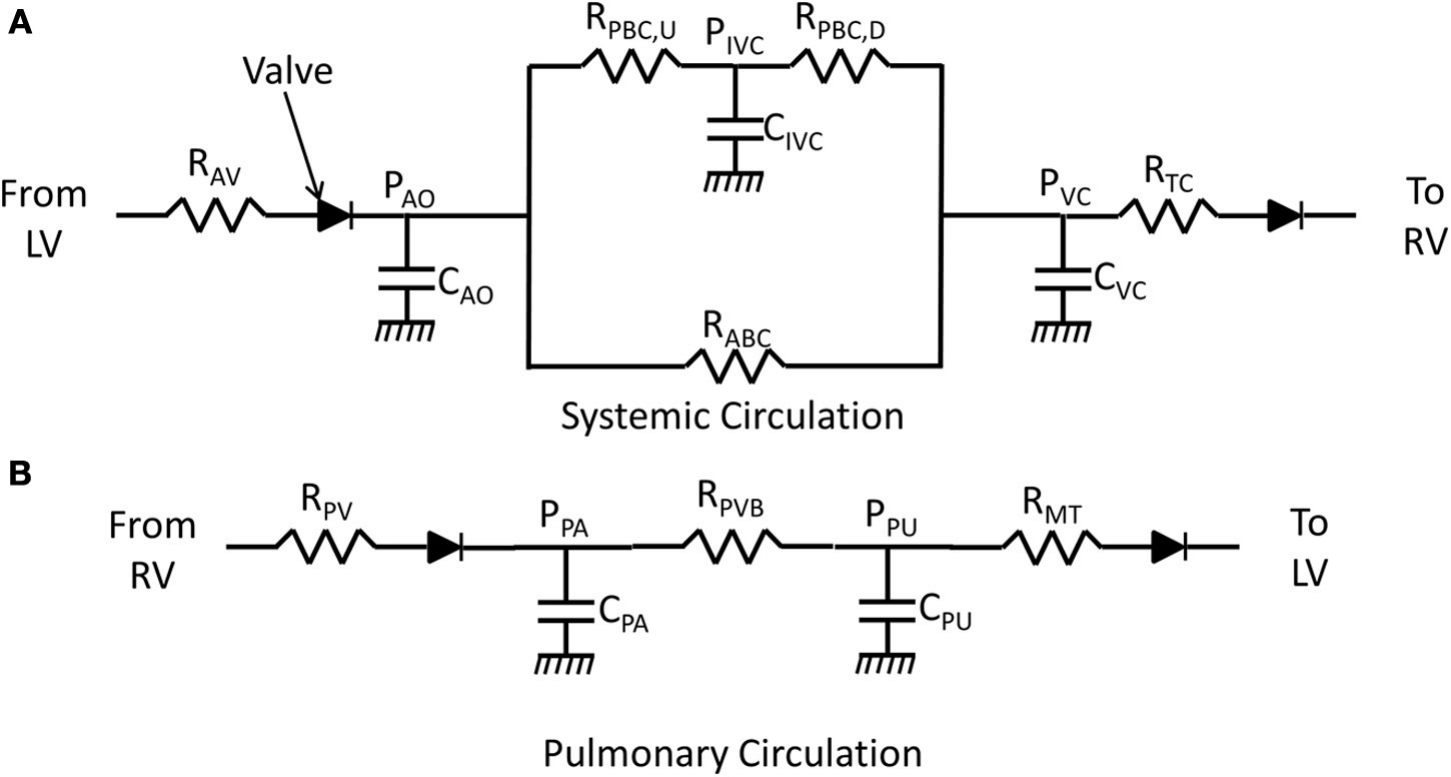
**Lumped parameter model of the circulation**. *R* represents resistance experienced by blood flowing through arteries/veins or valves. *C* represents compliance of an elastic chamber. *P* represents pressure in an elastic chamber. **(A)** Model of the systemic circulation (AV, aortic valve; AO, aorta; ABC, anterior-body circulation which drains into the superior vena cava; PBC, posterior body circulation which drains into inferior vena cava; IVC, inferior vena cava upstream of occlusion; VC, vena cava; TC, tricuspid valve). The compliance *C*_*VC*_ represents the compliance of the superior vena cava and the compliance of the inferior vena cava that is downstream of the site of occlusion. **(B)** Blood flow through the pulmonary vascular bed (PV, pulmonic valve; PA, pulmonary artery; PVB, pulmonary vascular bed; PU, pulmonary vein; MT, mitral valve).

The lumped parameter model used to represent the circulation is shown in Figure 1. In order to simulate an occlusion of the IVC, the systemic circulation is split into two parallel pathways, one representing the anterior body circulation that drains into the vena cava (VC), one representing the posterior body circulation draining first into the IVC and then into VC. The IVC compartment was added to capture the RV volume overshoot seen in the experiments. The pulmonary circulation is made up of two elastic compartments representing pulmonary arteries (PA) and pulmonary veins (PU). The resistances, labeled *R*, simulate the resistance experienced by blood passing through arteries/veins. The diodes represent the one-way valves at the inlet and exit of the ventricles; the valves also have resistance associated with them. The flow between compartments does not account for inertia and is calculated (Smith et al., 2004):

(1) $$Q=\frac{P_{1}-P_{2}}{R},$$

where the flow rate (*Q*) from compartment 1 to 2 is calculated from pressure in compartment 1 (*P*_1_) and compartment 2 (*P*_2_) and the resistance between the two compartments (R). The rate at which volume of a compartment changes is given by:

(2) $$\frac{dV}{dt}=Q_{\text{in}}-Q_{\text{out}}.$$

Assuming a linear relationship between pressure and volume, pressure in a compartment is calculated as:

(3) $$P_{\text{x}}=\left(V_{\text{x}}-Vd_{\text{x}}\right)C_{\text{x}},$$

where C_*x*_ is the compliance and Vd_*x*_ represents an unstressed volume of compartment *x*. Subscripts on pressure, elastance, and volume variables and parameters specify the model compartment with which each elastance, pressure, and volume is associated. These subscripts are defined in the legend of Figure 1.

The VCO perturbation is captured in the model by increasing the resistance *R*_PBC, D_ during the occlusion period by an amount determined by the adjustable parameter *pVCO* which represents the percentage of occlusion. Specifically, during the period of occlusion the resistance *R*_PBC, D_ is increased by a factor 1/(1-*pVCO*). When the occlusion is released, the resistance is returned to the non-occlusion value.

### Parameter estimation

The set of parameter values, δ, representing the control (0-day) group, is obtained using simultaneous least squares fitting of the pressure and volume recordings of all data from this group:

(4) $$\underset{\delta }{\min}E(\delta ),E(\delta )=\sum_{\forall X}\frac{\left(X_{\text{data}}-X_{\text{model}}(\delta )\right)}{\max(X)-\min{\left(X\right)}^{2}}.$$

Here *X* represents the variables RV pressure, RV volume, and aortic pressure. The minimization of the mean residual error (objection function) *E*(δ) for optimal estimation of the model parameter set is carried out using MATLAB (The MathWorks, Natick, MA) based code of FORTRAN package called PIKAIA implementing Genetic Algorithm (Charbonneau, 2002).

The control group values of all parameters, listed in Table 1, were estimated for each individual animal. From this analysis (see results and Table 1), 14 of the model parameters (indicated in Table 1) varied less than 5% within the group. It was therefore possible to set the values of these 14 parameters to the mean values from the control group and obtain reasonable fits to the experimental data for individuals from all groups. This set of parameters is denoted “globally fixed” in Table 1. The remaining 12 parameters, indicated “individually estimated” are estimated for each individual from each group.

**Table 1:** **Estimated parameters for 0-day hypoxic mice (*n* = 4)**.

**Parameter**	**Description**	**Value**	**Unit**
**TriSeg PARAMETERS**			
*A*^LW^_m, ref_ ^*^	Reference mid-wall surface area of LW	0.59±0.07	cm^2^
*A*^SW^_m, ref_ ^*^	Reference mid-wall surface area of SW	0.10±0.01	cm^2^
*A*^RW^_m, ref_^*^	Reference mid-wall surface area of RW	0.50±0.01	cm^2^
*v*_max_ ^*^	Sarcomere contractile velocity with no load	12.36±0.8	μ m/s
τ_D_^‡^	Factor scaling contraction decay time	53.6±2.9	ms
τ_R_^‡^	Factor scaling contraction rise time	76.6±2.4	ms
τ_SC_^‡^	Factor scaling duration of contraction	437.1±10.9	ms
**CIRCULATORY PARAMETERS**			
*R*_MT_^‡^	Mitral-valve resistance	3.56±0.02	mmHg·s/mL
*R*_AV_^‡^	Aortic-valve resistance	0.013±0.0001	mmHg·s/mL
*R*_TC_^‡^	Tricuspid-valve resistance	0.019±0.0001	mmHg·s/mL
*R*_PV_^‡^	Pulmonic-valve resistance	12.024±0.12	mmHg·s/mL
*R*_PVB_^*^	Pulmonary vascular bed resistance	154.32±11.1	mmHg·s/mL
*R*_ABC_^*^	Anterior body circulation resistance	1442.6±86.2	mmHg·s/mL
*R*_PBC_^†^^*^	Posterior body circulation resistance	569.6±32.9	mmHg·s/mL
*Vd*_VC_^*^	Unstressed vena cava volume	0.45±0.04	mL
*Vd*_IVC_^‡^	Unstressed IVC volume	1.04±0.05	mL
*Vd*_PA_^*^	Unstressed pulmonary artery volume	0.16±0.01	mL
*Vd*_PU_^‡^	Unstressed pulmonary vein volume	0.42±0.03	mL
*Vd*_AO_^‡^	Unstressed aorta volume	0.027±0.001	mL
*E*_VC_^*^	Vena-cava elastance	103.8±6.6	mmHg/mL
*E*_IVC_^‡^	IVC elastance	80.02±4.3	mmHg/mL
*E*_PA_^*^	Pulmonary artery elastance	437.78±26.6	mmHg/mL
*E*_PU_^‡^	Pulmonary vein elastance	188.5±10.2	mmHg/mL
*E*_AO_^‡^	Aorta elastance	3634.3±91.7	mmHg/mL
**PREDICTED IVC FLOW AND ESTIMATED VCO%**			
*IVC Flow*	IVC flow percentage	71.6±0.02	–
*pVCO*^║^^*^	Percentage of IVC occluded	88.6±1.4	–

‡ Globally fixed parameters.

* Individually estimated parameters.

† R_PBC_ represents total posterior body circulation resistance. The resistances upstream and downstream to inferior vena cava compliance (C_IVC_) are constrained to have a 90:10 (R_PBC, U_ : R_PBC, D_) ratio.

║ Represents percent occlusion downstream to inferior vena cava compliance (C_IVC_).

The individually estimated set of parameters (*A*^LW^_m, ref_, *A*^SW^_m, ref_, *A*^RW^_m, ref_, *v*_max_, *R*_PVB_, *E*_PA_, *Vd*_PA_, *R*_ABC_, *R*_PBC_, *E*_VC_, *Vd*_VC_, *pVCO*) includes 4 parameters associated with the heart model and 8 associated with the circulatory model. Three members of this set are associated with the pulmonary circulation: *R*_PVB_, *E*_PA_, *Vd*_PA_.

A significant increase in hematocrit levels is observed in the mice subjected to HySu compared to control (Wang et al., 2013). The observed increase in hematocrit from 48 ± 1 in the control group to ~70 in the PAH groups is expected to be associated with a substantial increase in blood viscosity. Associated effects on the effective resistances in the circulation are accounted for in the estimated resistance values.

For all parameters, two-tailed student's *t*-test was performed to compare the differences between the control group and the diseased group (14, 21, and 28-day). All results are represented as mean ± standard deviation (shown in Tables 1, 2). A *p* < 0.05 is considered as statistically significant. A list of all the parameters for the four groups can be found in section 3 of the supplementary material.

**Table 2:** **Parameters estimated to represent HySu dependent changes in model**.

**Parameter**	**0-day (*n* = 4)**	**14-day (*n* = 6)**	**21-day (*n* = 8)**	**28-day (*n* = 7)**
**TriSeg PARAMETERS**				
*A*^LW^_m, ref_	0.59±0.07	0.59±0.09	0.60±0.06	0.61±0.08
*A*^SW^_m, ref_	0.10±0.01	0.12±0.02	0.11±0.01	0.11±0.01
*A*^RW^_m, ref_	0.50±0.01	0.57±0.04^*^	0.59±0.05^*^	0.60±0.03^*^
*v*_max_	12.36±0.8	14.4±4.2	14.1±3.1	15.2±3.8
**CIRCULATORY PARAMETERS**				
*R*_PVB_	154.32±11.1	286.52±57.2^*^	265.5±43.4^*^	356.6±37.6^*^
*E*_PA_	437.78±26.6	560.3±101.7	622.5±172.1	938.6±161.6^*^
*Vd*_PA_	0.16±0.01	0.12±0.028	0.121±0.031	0.048±0.03^*^
*R*^†^_ABC_	1442.6±86.2	1946±394.3	1769.2±271.1	1883.3±453.2
*R*_PBC_^†^	569.6±32.9	804.3±121.9^*^	644.3±63.5	741±61.8^*^
*E*_VC_	103.8±6.6	103.8±25.3	123.0±45.5	69.8±16.2^*^
*Vd*_VC_	0.45±0.04	0.40±0.07	0.43±0.03	0.49±0.05
*pVCO*	88.6±1.4	81.21±0.07	80.8±0.04^*^	89.6±0.03

† These resistances were increased from their baseline values (shown in Table 1) to account for blood hematocrit increase in hypoxic mice.

* Indicates rejection of null-hypothesis at 5% significance level using two-tailed student's t-test with p <0.05 vs. 0-day hypoxic mice.

## Results

### Normoxia simulations

The control parameter (or baseline parameter) set is estimated from 4 independent control mouse recordings of RV pressure, volume, and aortic pressure. Heart rate is calculated from the aortic pressure time series to drive the sarcomere mechanics model (see section 1 of the supplementary material for details), which in turn modifies excitation-contraction of heart on a beat-to-beat basis.

**Figure 2:**
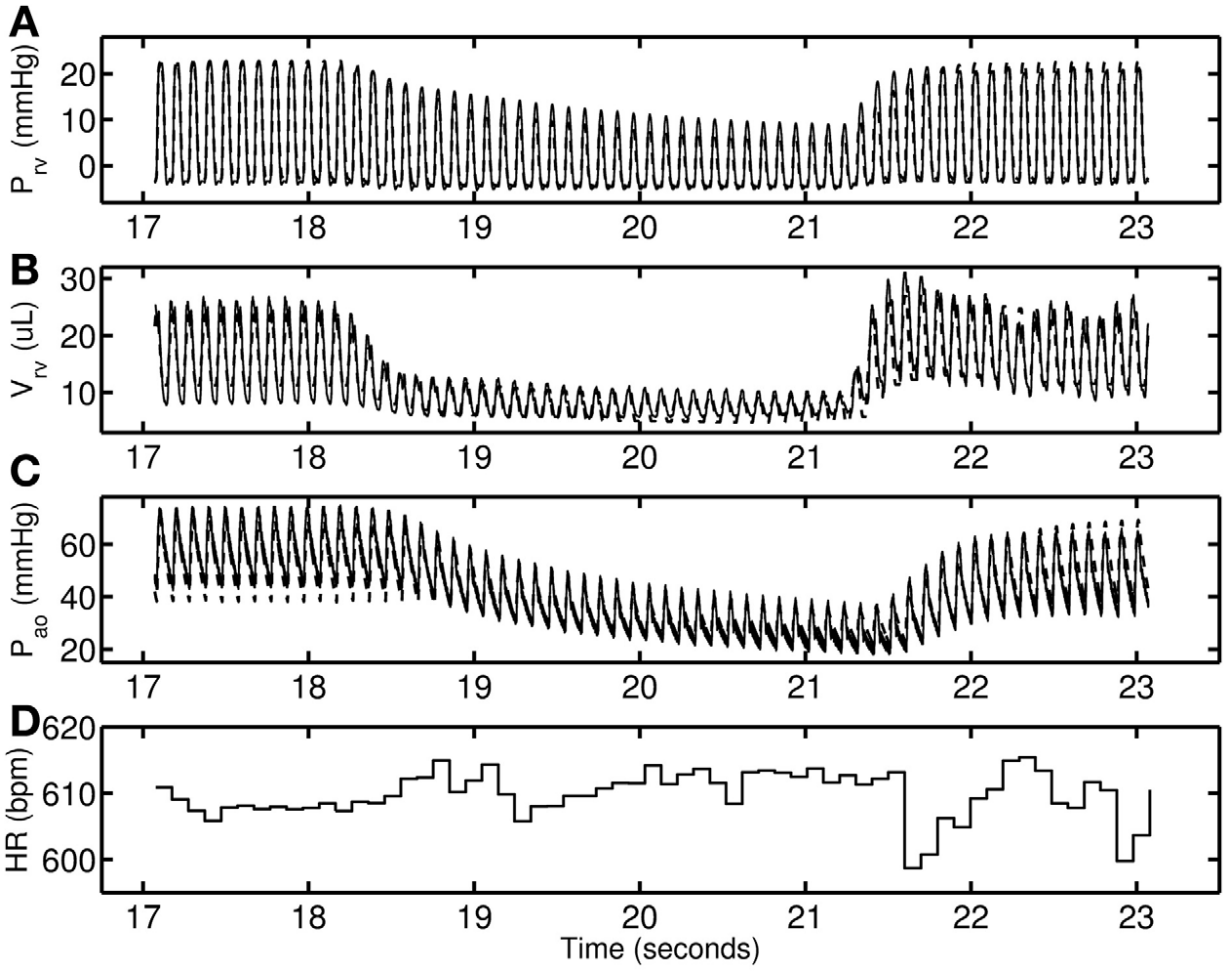
**A representative 0-day hypoxic mice data set (thin solid line) used for determining control parameter estimates**. The model simulations are shown as dashed line. **(A–C)** RV pressure, RV volume, and Aortic pressure. **(D)** Heart-rate in beats per minute used to modify excitation—contraction of heart on a beat-to-beat basis.

Figure 2 shows a representative data set from the control group. The RV pressure and volume decreases with VCO. Following the release of the occlusion there is an overshoot in RV volume (Figure 2B) that is effectively captured by the model. In the model this phenomenon follows from interaction between the IVC compliance upstream to the point of occlusion; see Figure 1) and VC compliance (downstream to the point of occlusion; see Figure 1). During the occlusion, the vasculature upstream of the occlusion increases in volume and the release of the accumulated fluid drives the overshoot in RV filling. The degree of overshoot is due to interaction between the vena cava compliances upstream (C_IVC_) and downstream (C_VC_) of the point of occlusion (R_PBC, D_). Heart rate remains with ±0.7% of the mean value for the time course illustrated in Figure 2. The lack of an increase in heart rate during the period of occlusion indicates that the baroreflex is effectively suppressed by the anesthetic, as baroreflex fibers are expected to respond to a pressure drop within, as soon as, a single beat (Coleridge et al., 1984).

**Figure 3:**
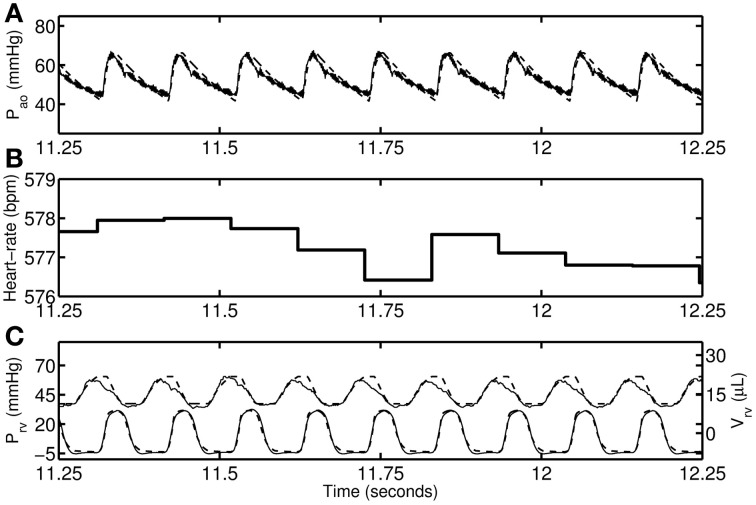
**Baseline RV pressure, RV volume, and aortic pressure of a representative 14-day hypoxic mice shown for 1-s duration. (A)** Aortic pressure recording (thin solid line) and model simulation (thick dashed line). **(B)** Heart-rate during the period. **(C)** RV pressure data (thin solid line; lower graph) and model (thick dashed line), and RV volume data (thin solid line; upper graph) and model (thick dashed line).

Figure 3 shows a detail of the data and model simulations from a representative 14-day mouse, illustrating the steady conditions before occlusion. Representative comparisons of experimental data and model simulations for each experimental group individual are provided in section 2 of the supplementary material.

### IVC occlusion and release

**Figure 4:**
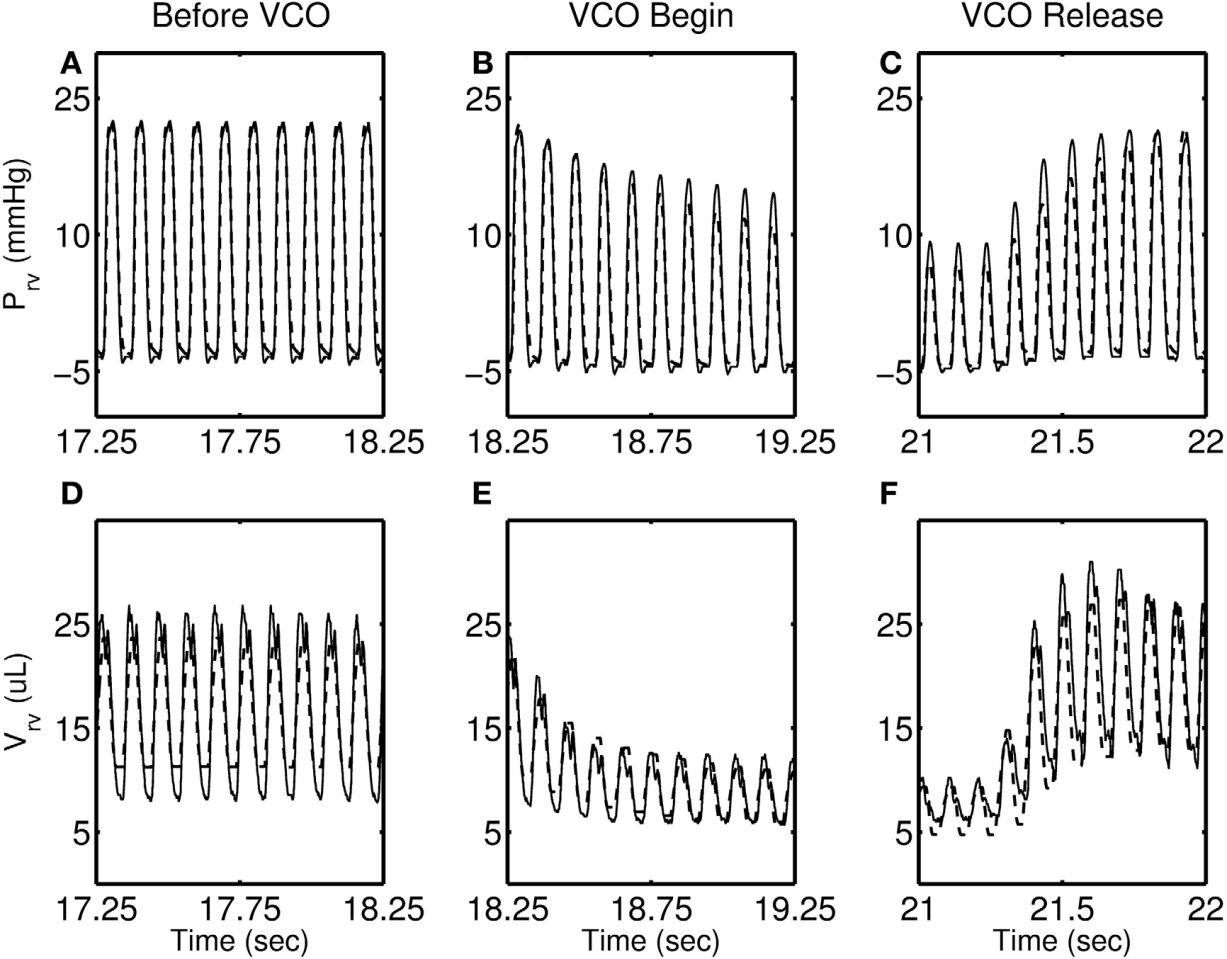
**RV pressure (A–C) and RV volume (D–F) data (thin solid line) with model simulations (thick dashed line) shown for a representative 0-day hypoxic mice**.

**Figure 5:**
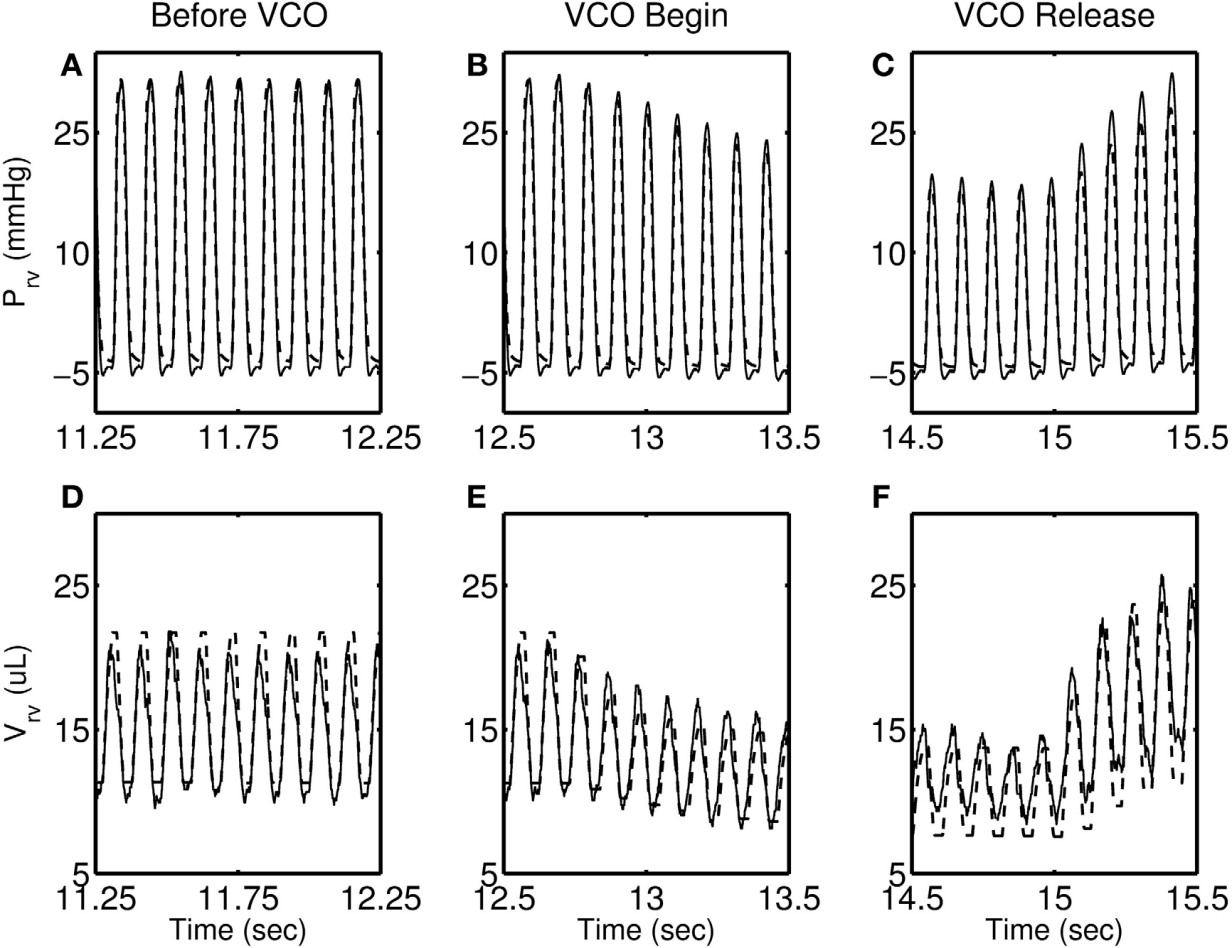
**RV pressure (A–C) and RV volume (D–F) data (thin solid line) with model simulations (thick dashed line) shown for a representative 14-day hypoxic mice**.

**Figure 6:**
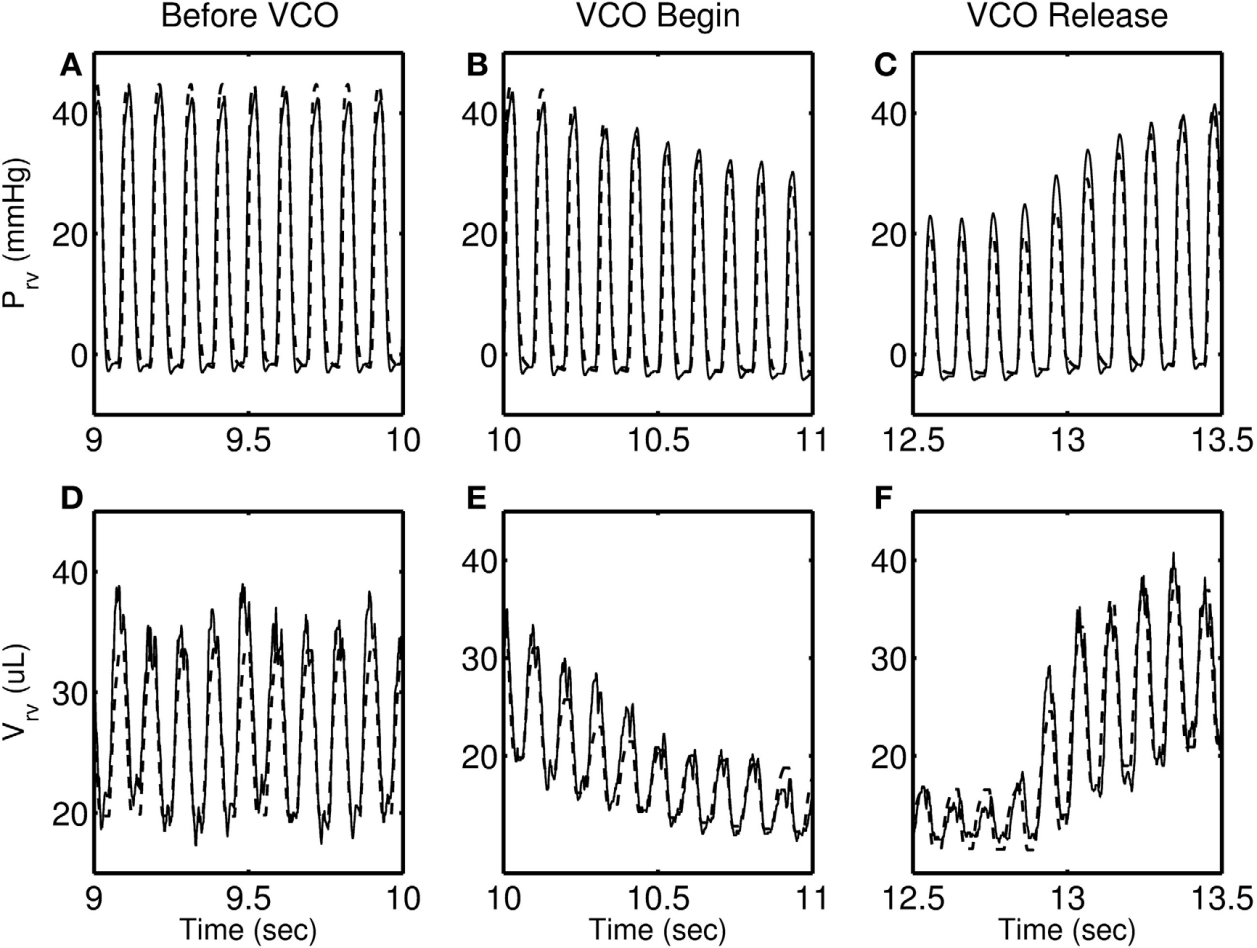
**RV pressure (A–C) and RV volume (D–F) data (thin solid line) with model simulations (thick dashed line) shown for a representative 21-day hypoxic mice**.

**Figure 7:**
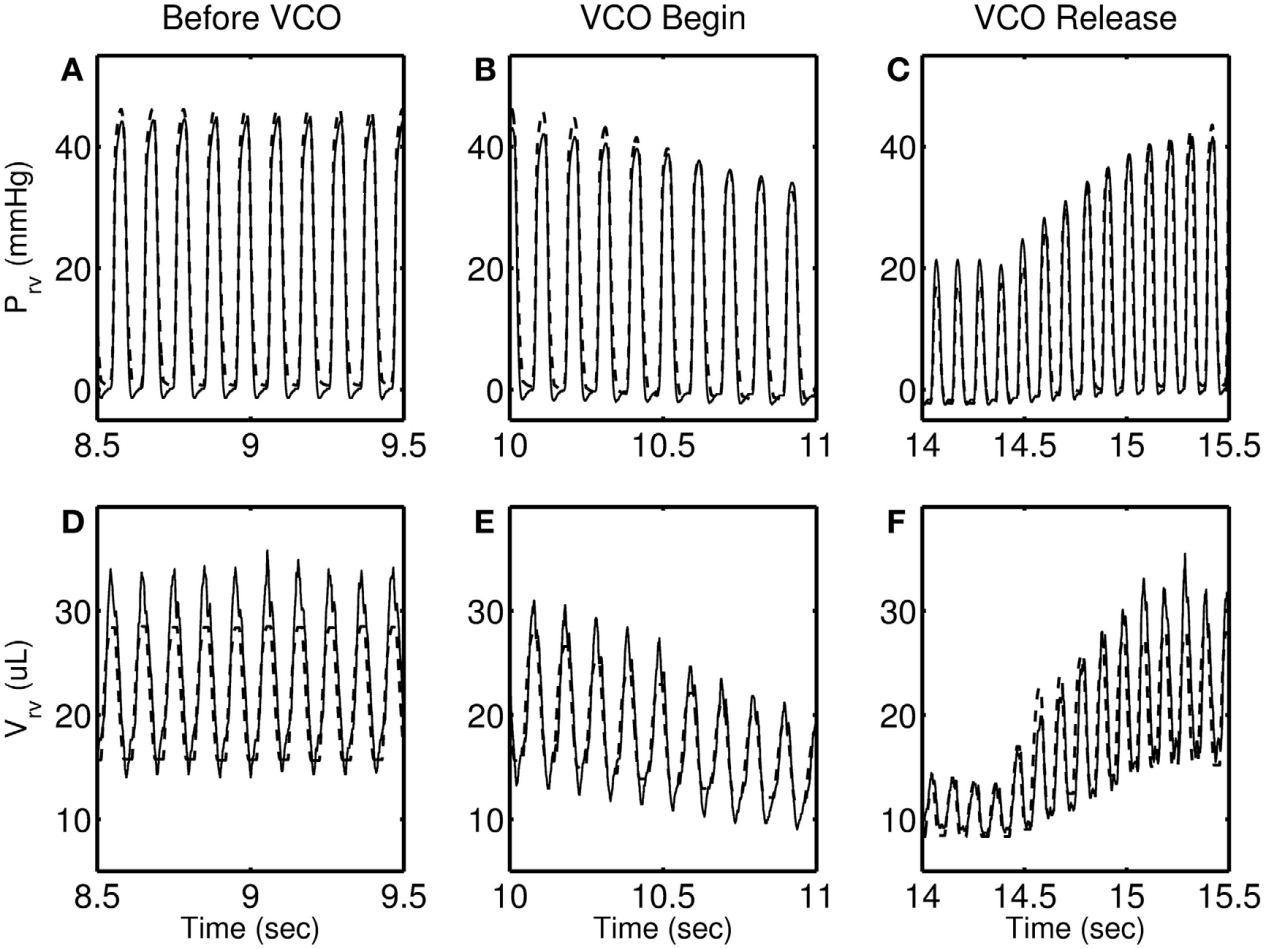
**RV pressure (A–C) and RV volume (D–F) data (thin solid line) with model simulations (thick dashed line) shown for a representative 28-day hypoxic mice**. Please note that the time taken by RV pressure (and volume) to its baseline (panels **C** and **F**) is longer than 0, 14, and 21-day individuals.

Figures 4–7 show model simulation and experimental data for a representative individual from each group during baseline (steady state), beginning of occlusion and release of occlusion. To effectively illustrate behavior during key periods of the experimental protocol, these figures illustrate one second duration of baseline (before VCO), the first second following occlusion (VCO begin), and one second of data around the occlusion release (VCO release). In all cases, the model is able to capture the key circulatory dynamics and the increase in baseline RV pressure (Figures 4A, 5A, 6A, 7A) and end-diastolic volume (Figures 4B, 5B, 6B, 7B) associated with chronic exposure to HySu.

**Figure 8:**
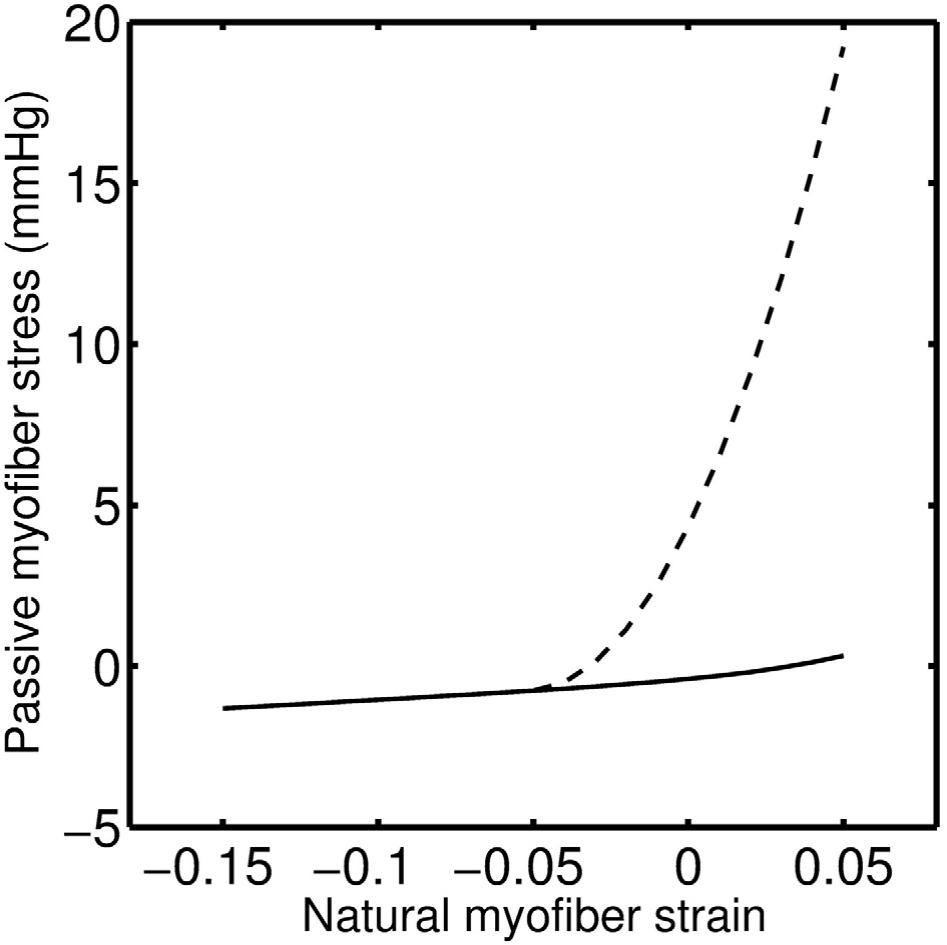
**Modified myofiber stress-strain relationship for the RW mechanics of a 28-day hypoxic individual (dashed line) as compared to 0, 14, and 21-day individuals (solid line)**. Zero myofiber strain corresponds to reference sarcomere length of 2.0 μm. Positive strain is following tensile stress and negative under compressive stress.

Following VCO release, the RV pressure and volume return to their baseline values within a few heart beats for 0, 14, and 21-day hypoxic mice (see Figures 4C,F, 5C,F, 6C,F). However, for 28-day hypoxic mice, RV pressure (and volume) takes several beats to return to the baseline values (see Figures 7C,F). This phenomenon may be related to an increase in the passive stiffness of the RV free wall. Indeed, it was found that the model could not effectively capture the experimental observation for the 28-day group based on varying only the parameters listed in Table 2. For this group, it was necessary to adjust the passive myofiber stress-strain curve used in the TriSeg heart model. Figure 8 shows the passive stress-strain relationship obtained from the default parameterization of the TriSeg model (solid curve). This parameterization was used for all 0, 14, and 21-day simulations. To match the data for the 28-day group, RV free wall passive stiffness was increased by lowering the transition point of passive stress generation, which is captured in the first term of the empirical passive stress-strain relationship (For details see section 1 of the supplementary material). This predicted increase in passive stiffness is consistent with the increase in RW collagen accumulation found by Wang et al. (2013). This proposed explanation of the 28-day data does not exclude other explanations, such as remodeling of RV and septal geometry that is not fully captured by our model.

## Discussion

**Figure 9:**
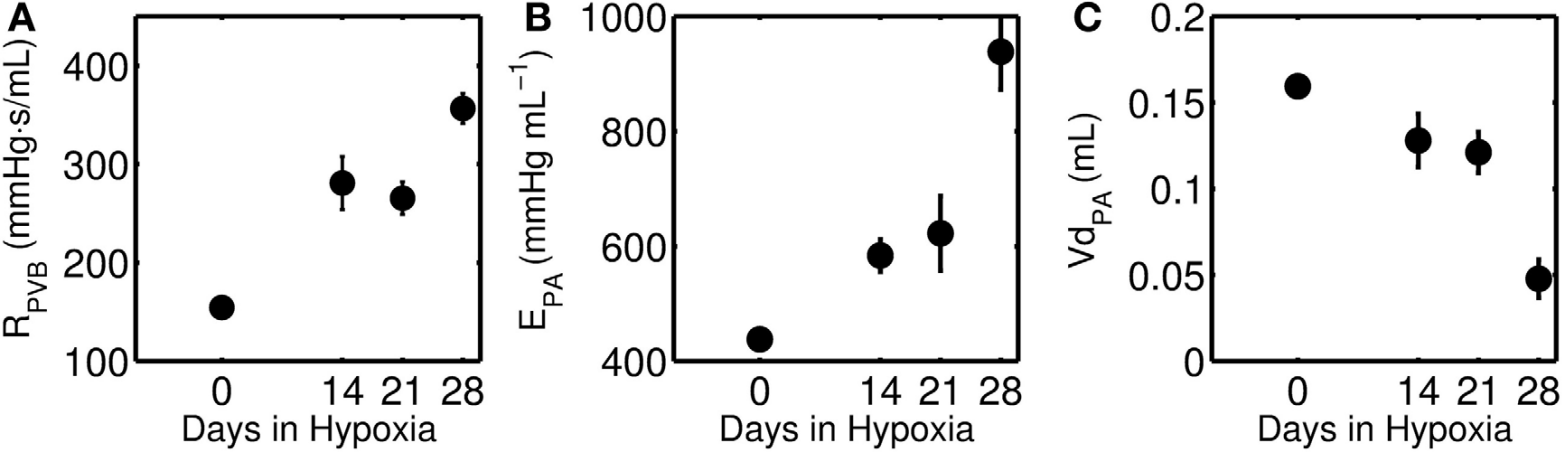
**(A)** Pulmonary vascular resistance (R_PVB_), **(B)** Pulmonary artery elastance (E_PA_), and **(C)** unloaded pulmonary artery volume (Vd_PA_) as a function of increasing days of hypoxia and treatment with SUGEN. The closed circles represent mean (with standard error) taken over 0-day (*n* = 4), 14-day (*n* = 6), 21-day (*n* = 8), and 28-day (*n* = 7) hypoxic mice group. The decrease in Vd_PA_ and increase in E_PA_ is statistically significant only for the 28-day group (*p* < 0.001).

It is known that PAH is associated with structural remodeling of the pulmonary circulation resulting in increased pulmonary vascular resistance (Stenmark and McMurtry, 2005; Wang and Chesler, 2011). Based on model fitting of experimental data obtained from a mouse model of PAH, pulmonary vascular resistance (R_PVB_), pulmonary arterial elastance (E_PA_), and pulmonary arterial narrowing (Vd_PA_) were estimated as functions of PAH progression (see Figure 9). This analysis suggests monotonic changes in these parameters as PAH progressed, with ~130% and ~110% increases in R_PVB_ and E_PA_ after 28 days of PAH (Estimated increases in both of these parameters are statistically significant after 28-days of HySu, *p* < 0.001). The predicted increase in *R*_PVB_ is consistent with the experimental measurement on total PVR by Wang et al. (2013). The changes in *E*_PA_ suggest a continuous stiffening of the pulmonary vascular bed with PAH development, which is as expected but cannot be obtained directly from the *in vivo* pressure-volume loops. It is known that pulmonary vascular resistance and pulmonary arterial elastance are major contributors to the increased RV afterload, and consequently RV failure (Naeije and Huez, 2007). To delineate the individual contributions of *R*_PVB_ and *E*_PA_, we performed model simulations with either *R*_PVB_ or *E*_PA_ or both set to 1.5 times their control values (see section 4 of the supplementary material). Model simulations reveal that increased *R*_PVB_ and *E*_PA_ individually contribute more toward increased afterload and preload, respectively. However, the increases in afterload and preload are more prominent when *R*_PVB_ and *E*_PA_ are increased together (see section 4 of the supplementary material).

Abe et al. (2010) reported that HySu exposure in rat leads to progressive pulmonary hypertension and vascular remodeling. Predicted changes in unstressed volume Vd_PA_ and pulmonary arterial elastance E_PA_ are not statistically significant for the 14-day group. Similarly, the predicted reduction in Vd_PA_ and increase in E_PA_ for the 21-day group are not statistically significant. However, the decrease in Vd_PA_ and increase in E_PA_ are statistically significant for the 28-day group (*p* < 0.001), suggesting significant pulmonary vascular remodeling at this stage in response to HySu.

It has been reported that the increased afterload associated with chronic hypoxia exposure alone will lead to RV functional adaptation to maintain ventricular-vascular coupling (Wauthy et al., 2004). However, a more severe stage of PAH associated with sustained increase in afterload will lead to the ventricular-vascular decoupling and RV failure (Lankhaar et al., 2006). The mechanism for RV changes from adaptation to dysfunction or failure at different stages of PAH remains largely unknown. In this modeling study, the failure of the normotensive passive stress-strain relationship, for the RW, to explain the data from the 28-day group suggests a substantial increase in passive myocardial stiffness after 28-day of HySu and possibly the transition to dysfunction or failure.

**Figure 10:**
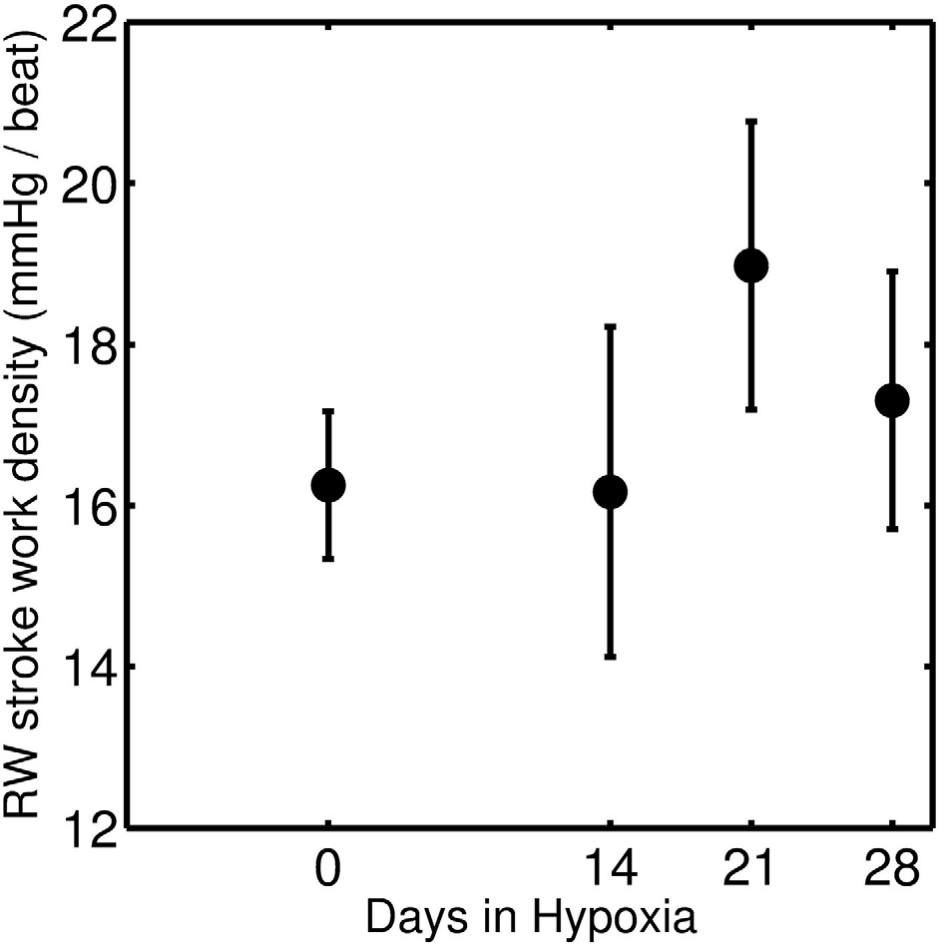
**Stroke work density of RW which is the area of the myofiber stress-strain curve, and is defined as contractile myofiber work per unit of tissue volume per beat**. The closed circles represent mean (with standard error) taken over 0-day (*n* = 4), 14-day (*n* = 6), 21-day (*n* = 8), and 28-day (*n* = 7) hypoxic mice group.

To investigate the effect of HySu on mechanical load response of RV, the RW stroke work density (defined as contractile myofiber work per unit tissue volume) was calculated. Stroke work density is computed as the area enclosed by the myofiber stress-natural strain loop (Kroon et al., 2009; Lumens et al., 2009a):

(5) $$w_{f}=\oint \sigma_{f}d\varepsilon_{f},$$

where σ_*f*_ is Cauchy myofiber stress, ε_*f*_ is natural myofiber strain, and *w*_*f*_ is RW stroke work density. Calculated mean stroke work densities for the four groups are shown in Figure 10. Results indicate an increase in RW stroke work density for the 21-day group (compared to the control and 14-day groups) that may be indicative of increased contractility in response to increased afterload (Wauthy et al., 2004). This increase in RW stroke work density is followed by a decrease in the 28-day group, suggesting a pathological/maladaptive remodeling in response to sustained increase in afterload. However, due to a large degree of individual variability, these trends are not found to be statistically significant.

Wang et al. (2013) report RV stroke work and stroke work density for 0, 14, 21, and 28-day group individuals suggesting a monotonic increase in stroke work as a function of days of HySu exposure but no apparent change in stroke work density [See Figure 6 of Wang et al. (2013)]. There are several reasons why the mean of model predictions of stroke work density do not follow the same trend as those reported by Wang et al. (2013). First, the model tends to underestimate the peak diastolic volume compared to the data for individuals from the 28-day group (see Figure 7D and Figures S19–S25). Second, calculated stroke work density in Figure 10 corresponds solely to the contribution from the RW while work computed from the area of the pressure volume loop corresponds to the whole ventricle. Thus whether or not the model-predicted drop in work density for the 28-day group reflects a transition to RV dysfunction is not clear. Further analysis of more individuals and/or individuals with more severe disease phenotype may shed light on this question.

In sum, the major findings to emerge from this study are:

1. Observed increases in pulmonary vascular resistance associated with PAH are accompanied by commensurate increases in pulmonary arterial stiffness. Predicted changes in vascular resistance and elastance with chronic hypoxia and SUGEN treatment are illustrated in Figure 9, and tabulated in Table 2.
2. Model analysis predicts a significant inward remodeling of the pulmonary arteries that becomes apparent only in the later stage of PAH, i.e., 28-day group. Inward remodeling of the PA is captured by the parameter Vd_PA_, defining the volume of PA compartment at zero pressure. Thus the model prediction is that the volume of the PA compartment is significantly reduced in late stage PAH.
3. Model analysis predicts substantial stiffening of RV in the 28-day group, suggesting a degree of maladaptive remodeling in this group. While the fact that it was not necessary to increase the passive stiffness of the RV compared to the baseline to represent the 14- and 21-day groups does not necessarily mean changes in the passive mechanical properties of the myocardium have not occurred in these groups, it does demonstrate that any changes that did occur become apparent only in the 28-day group. This substantial increase in passive stiffness in the 28-day group is associated with a predicted drop in the mean of RV free wall stroke work density per beat, as illustrated in Figure 10.

### Conflict of interest statement

The authors declare that the research was conducted in the absence of any commercial or financial relationships that could be construed as a potential conflict of interest.
