# Supplementary material for: Analysis of cardiovascular dynamics in pulmonary hypertensive C57BL6/J mice
Source: Front Physiol. 2013 Dec 11;4:355. doi: 10.3389/fphys.2013.00355 (PMC3858724; doi:10.3389/fphys.2013.00355)
Supplement: Supplementary file 1 [file Presentation1.PDF]

Electronic Supplementary Material for the paper:

Analysis of cardiovascular dynamics in pulmonary hypertensive C57BL6/J mice

Shivendra G Tewari[1], Scott M Bugenhagen[1,2], Zhijie Wang[3], David A Schreier[3], Brian E Carlson[1,2], Naomi C Chesler[3], Daniel A Beard[1,2,#]

[1]Biotechnology & Bioengineering Center, [2]Department of Physiology, Medical College of Wisconsin, 8701 Watertown Plank Road, Milwaukee, WI 53226. [3]Department of Biomedical Engineering, University of Wisconsin–Madison, Madison, WI 53706.

#To whom correspondence should be addressed. E-mail: beardda@gmail.com

Section 1: Myofiber Mechanics Model

This section describes the heuristic model that converts natural myofiber strain, ε_f, to Cauchy myofiber stress, σ_f which is described in detail in (Lumens et al., 2009). This model is based on sarcomere contraction experiments on isolated rat cardiac muscle (ter Keurs et al., 1980;de Tombe and ter Keurs, 1990) but was scaled by Lumens et al. (Lumens et al., 2009) to simulate human ventricular mechanics and hemodynamics. The natural myofiber strain is converted to sarcomere length L_s by:

$$L_s = L_{s,\mathrm{ref}} e^{\varepsilon \mathrm{f}}, \tag{1}$$

where $L_{s,\mathrm{ref}}$ represent reference sarcomere length at zero strain.

The sarcomere model is based on Hill's three element muscle model (Hill, 1938).The time dependent behavior is simulated using two state variables, namely, L_{sc} (contractile element length) and C (mechanical activation). Physiologically the mechanical activation variable, C, relates to the intracellular calcium concentration. The rate of change of L_{sc} depends linearly on the length of series elastic element ($L_s - L_{sc}$):

$$\frac{dL_{\mathrm{sc}}}{dt} = \left(\frac{L_{\mathrm{s}} - L_{\mathrm{sc}}}{L_{\mathrm{se,iso}}} - 1 \right) v_{\max}, \tag{2}$$

here $v_{\max}$ represents sarcomere shortening velocity under no load and $L_{\mathrm{se,iso}}$ is the isometrically stressed series elastic element. The rate of change of C is heuristically determined with two separate terms describing rise and decay:

$$\frac{dC}{dt} = \frac{1}{\tau_{\mathrm{R}}} \cdot I_{\mathrm{L}}\left(L_{\mathrm{sc}}\right) \cdot R_{\mathrm{C}}(t) + \frac{1}{\tau_{\mathrm{D}}} \cdot \frac{C_{\mathrm{rest}} - C}{1 + e^{\left(D_{\mathrm{C}}(L_{\mathrm{sc}}) - t\right)/\tau_{\mathrm{D}}}}, \tag{3}$$

here τ_{R} and τ_{D} scale the rise and decay time of C; t and C_{rest} represent the time and the resting level of activation. The functions I_{L}, R_{C} and D_{C} represent increase, rise and decrease of activation respectively:

$$\begin{aligned} &I_{\mathrm{L}}(L_{\mathrm{sc}}) = \tanh\left(4.0(L_{\mathrm{sc}} - L_{\mathrm{sc0}})^2\right), \\ &R_{\mathrm{C}}(t) = 0.02 \cdot x^3 (8-x)^2 e^{-x} \text{ with } x = \min(8, \max(0, t/\tau_{\mathrm{R}})), \\ &D_{\mathrm{C}}(L_{\mathrm{sc}}) = \tau_{\mathrm{sc}}(0.29 + 0.3 L_{\mathrm{sc}}), \end{aligned} \tag{4}$$

where L_{sc0} represents contractile element length under no load and τ_{SC} is a factor scaling duration of contraction.

Beat-to-beat changes in mechanical activation variable of the sarcomere model are accounted by scaling the three time-constants by a factor $\alpha = HR_{\mathrm{human}} / HR_{\mathrm{mouse}}$, where HR_{human} is beats per second for human, chosen to be 1.17 beats per second $\left(= \frac{70 \text{ beats per minutes}}{60 \text{ minutes}} \right)$, and HR_{mouse} is the beats per second for an individual mouse derived from systemic pressure measurements.

Active myofiber stress, $\sigma_{\mathrm{f,act}}$, is calculated by the following expression which depends on the two state variables i.e. L_{sc} and C:

$$\sigma_{\mathrm{f,act}} = \sigma_{\mathrm{act}} \cdot C \cdot (L_{\mathrm{sc}} - L_{\mathrm{sc0}}) \cdot \frac{L_{\mathrm{s}} - L_{\mathrm{sc}}}{L_{\mathrm{se,iso}}}. \tag{5}$$

The expression for passive myofiber stress accounts for the effect of titin and collagen that provides a soft behavior for low myofiber strain and a stiff behavior for large positive strain:

$$\sigma_{\mathrm{f,pas}} = \sigma_{\mathrm{pas}} \left(36 \cdot \max(0, \varepsilon_{\mathrm{f}} - 0.1)^2 + 0.1(\varepsilon_{\mathrm{f}} - 0.1) + 0.0025 e^{30\varepsilon_{\mathrm{f}}}\right). \tag{6}$$

This relation assumes that stress-strain relation is dominated by titin at low strain levels while at high strain levels it is dominated by collagen fibers. In Eq. (6) the last term accounts for the effect of collagen and the second term accounts for the effect of titin. The first term makes the stress-strain relation steeper than the original exponential term beyond the transition point. In fact, it was this transition point (which in the above expression is 0.1) that was altered to account for the hypoxia (with SUGEN) significant remodeling observed in the 28-day mice. For 28-day mice, the stress-strain relationship shown in Eq. (6) reads as follows:

$$\sigma_{\mathrm{f,pas}} = \sigma_{\mathrm{pas}} \left(36 \cdot \max(0, \varepsilon_{\mathrm{f}} + 0.05)^2 + 0.1(\varepsilon_{\mathrm{f}} - 0.1) + 0.0025 e^{30\varepsilon_{\mathrm{f}}}\right). \tag{7}$$

All the parameters mentioned in this section have same values as reported initially by Lumens et al. (Lumens et al., 2009) except for the time constants shown in Eq. (3) and (4) which were estimated for 0-day mice, and are reported in Table 1 of the manuscript. Moreover, the analysis of experimental data suggested that left ventricle and septum contract 10 millisecond before right ventricle for which we accounted by subtracting a parameter, $\lambda = 10$ ms, from the variable t mentioned in Eq. (3) and (4) for equations governing left free wall and septal wall.

Section 2: Supplementary Figures

a. The TriSeg Model

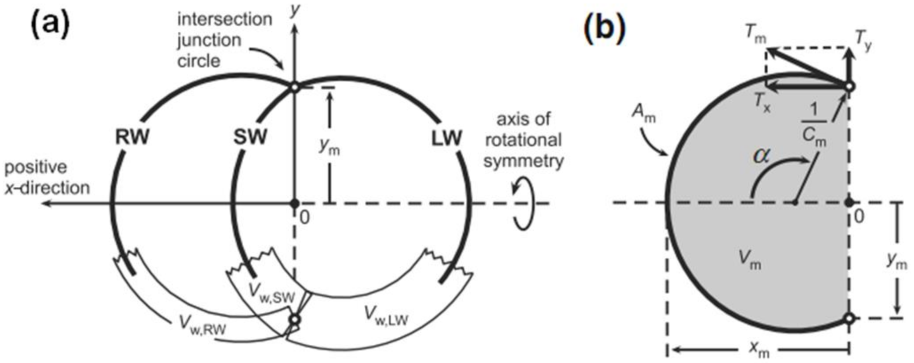

Figure S1: Adapted from Lumens et al. (Lumens et al., 2009). (a) Cross-section of the composite, forming the left and right ventricles, through the axis of rotational symmetry. Right free wall (RW), Septal wall (SW), and Left free wall (LW) intersect at the junction circle which is perpendicular to the plane of drawing (dotted lines). The center of the junction circle is the origin (0) with respect to which the coordinates are applied. The midwall surface divides the walls with volume V_w into two shells of equal volume. Note: The positive direction is applied towards RW. (b) Cross-section of a single midwall surface area through the axis of rotational symmetry. A_m represents the surface area of the midwall geometry enclosed between midwall surface and junction circle. V_m is the volume shown as the shaded region; C_m is the reciprocal of radius of curvature. α is the half-open angle with respect to which the transmural pressure (T_m) is resolved in an axial (T_x) and radial (T_y) component. (Figure reproduced from Lumens et al. (Lumens et al., 2009), permission pending.)

b. <u>Experimental Data v/s Model Simulations of other 0-day mice</u>

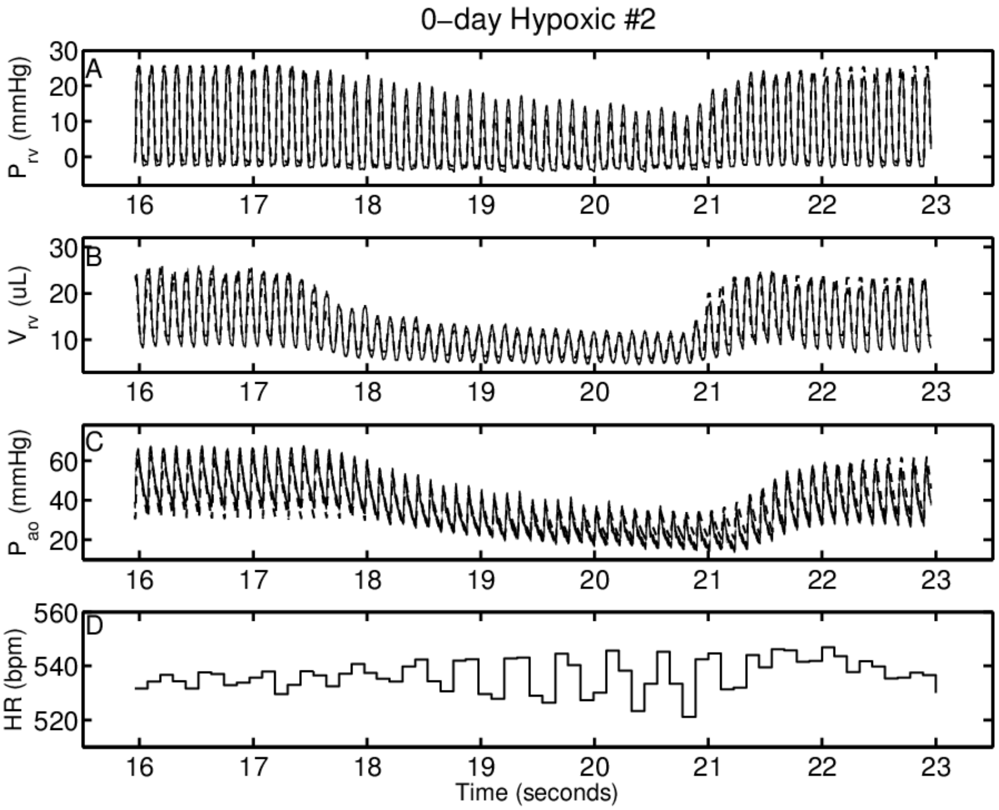

Figure S2:

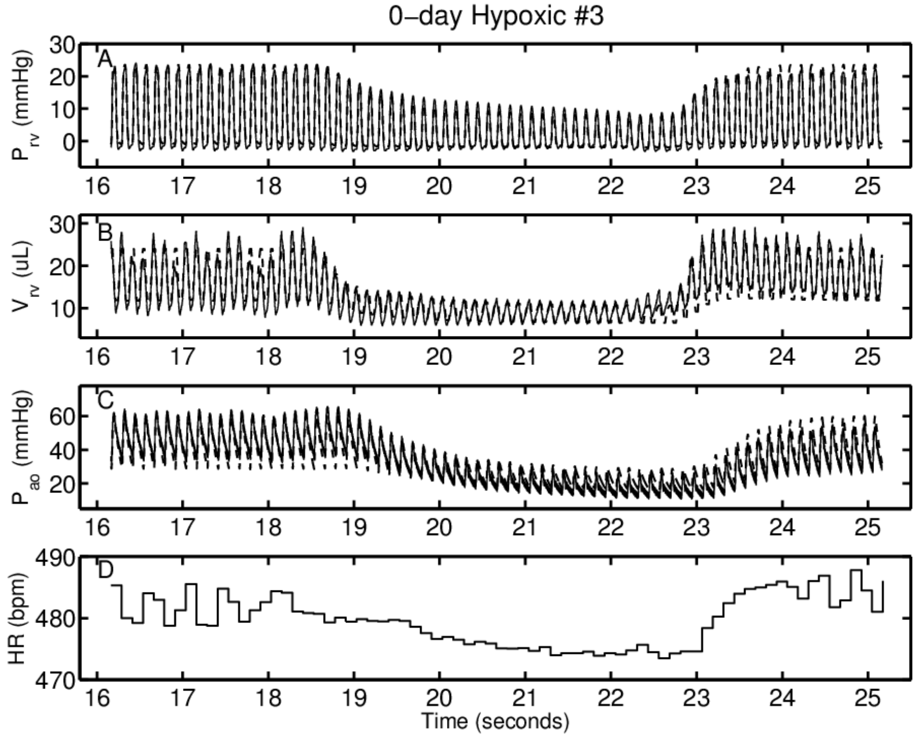

Figure S3:

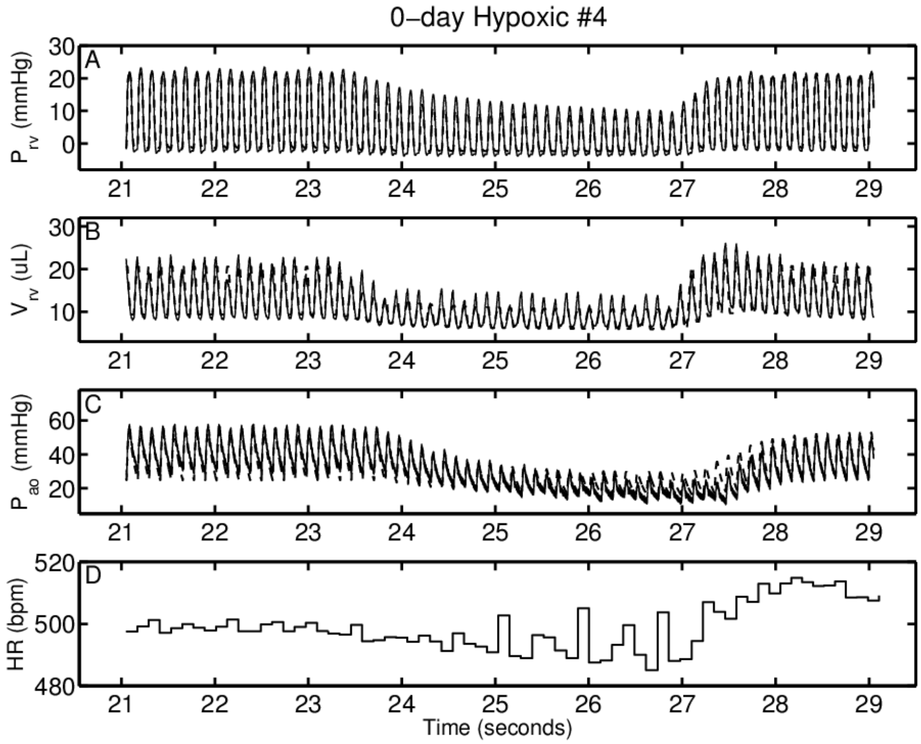

Figure S4:

c. Experimental Data v/s Model Simulations of 14-day mice

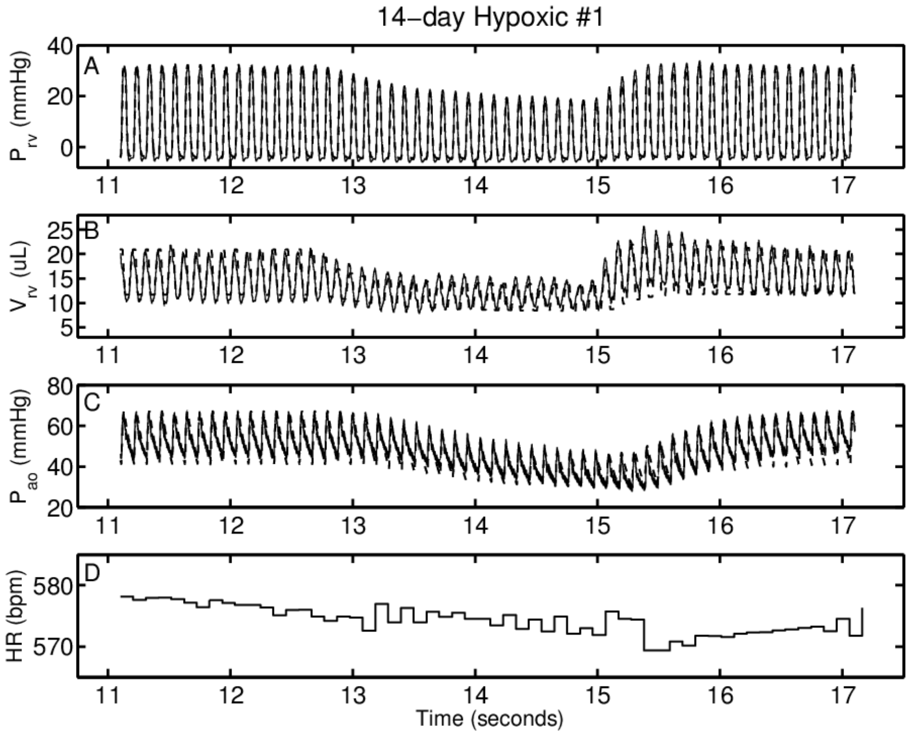

Figure S5:

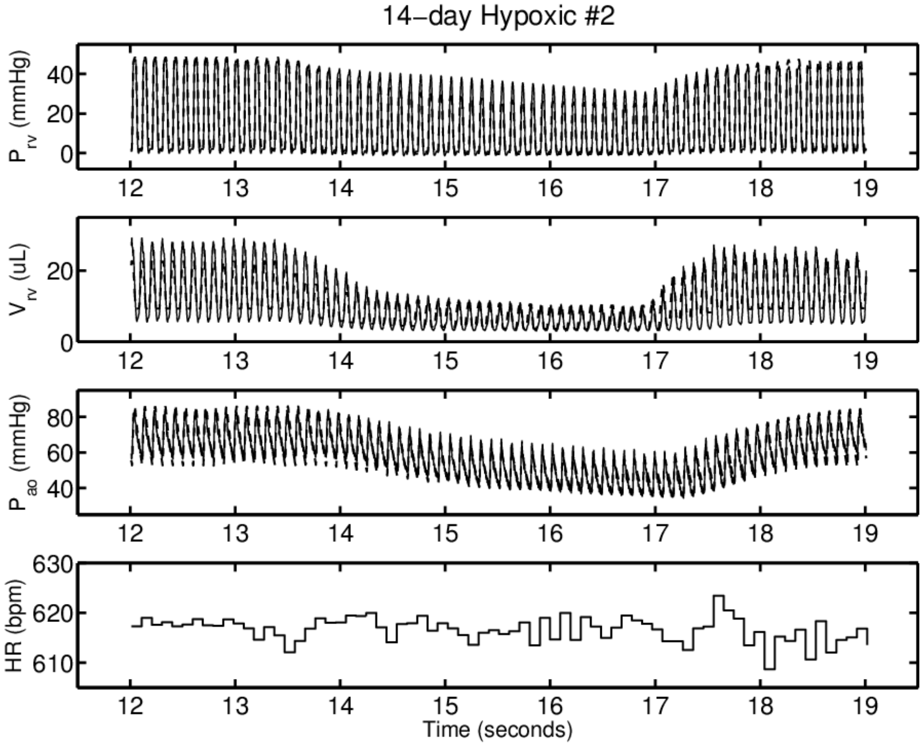

Figure S6:

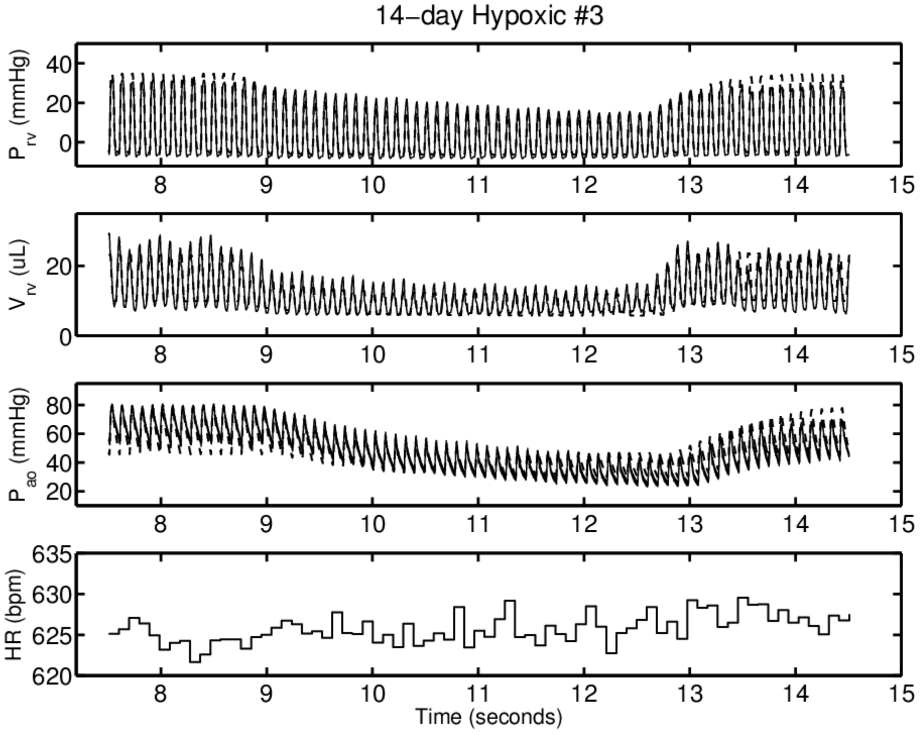

Figure S7:

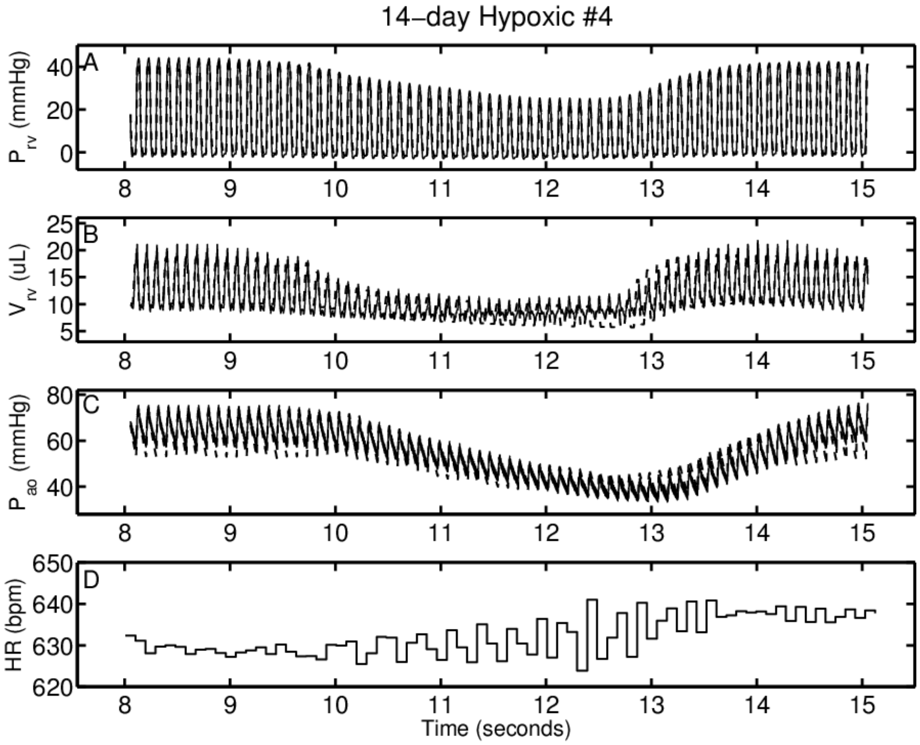

Figure S8:

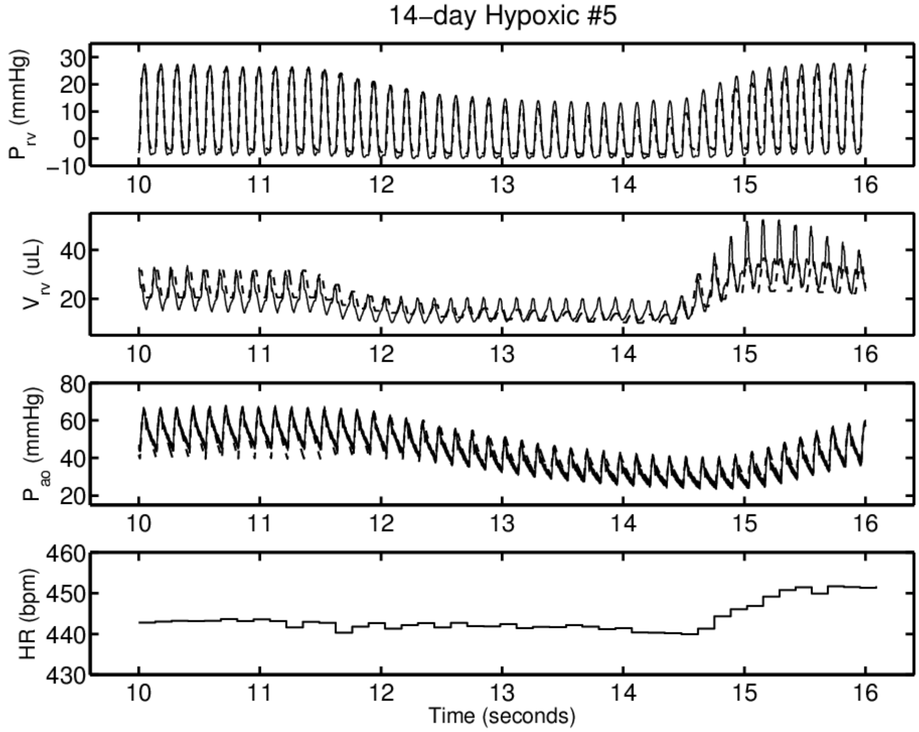

Figure S9:

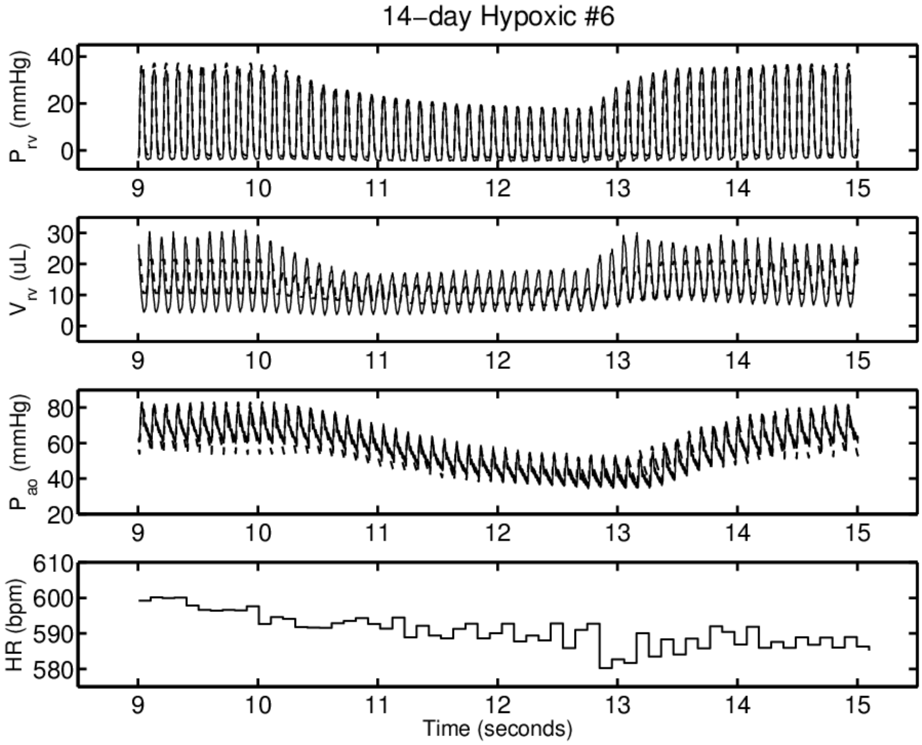

Figure S10:

d. Experimental Data v/s Model Simulations of 21-day mice

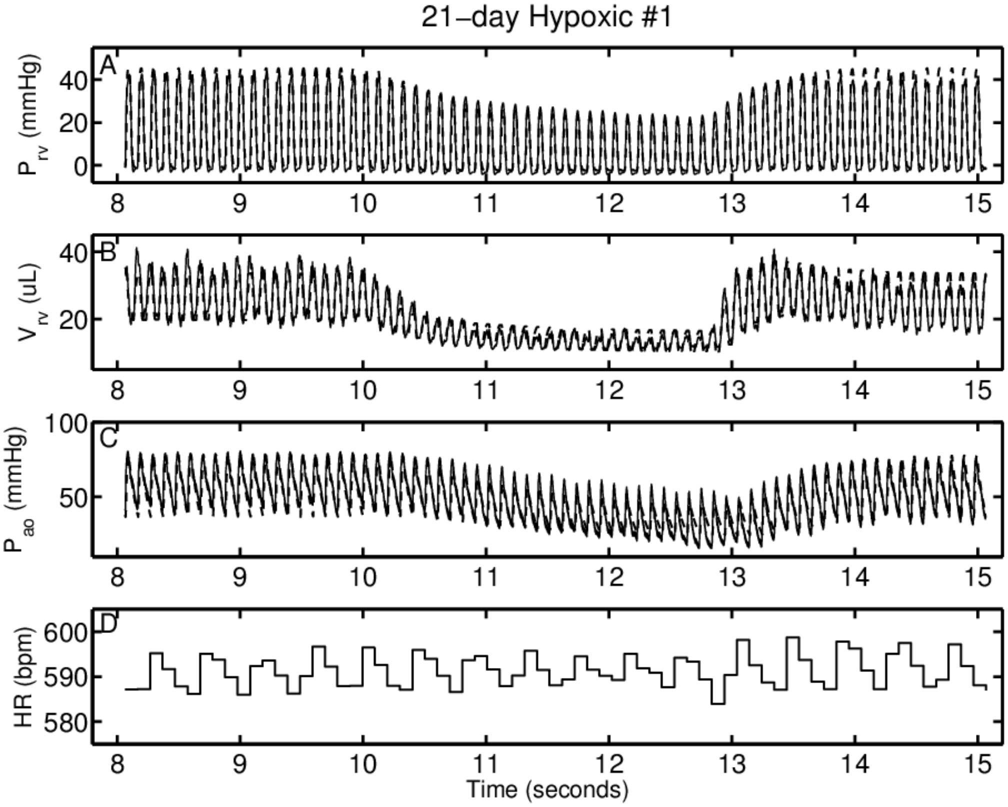

Figure S11:

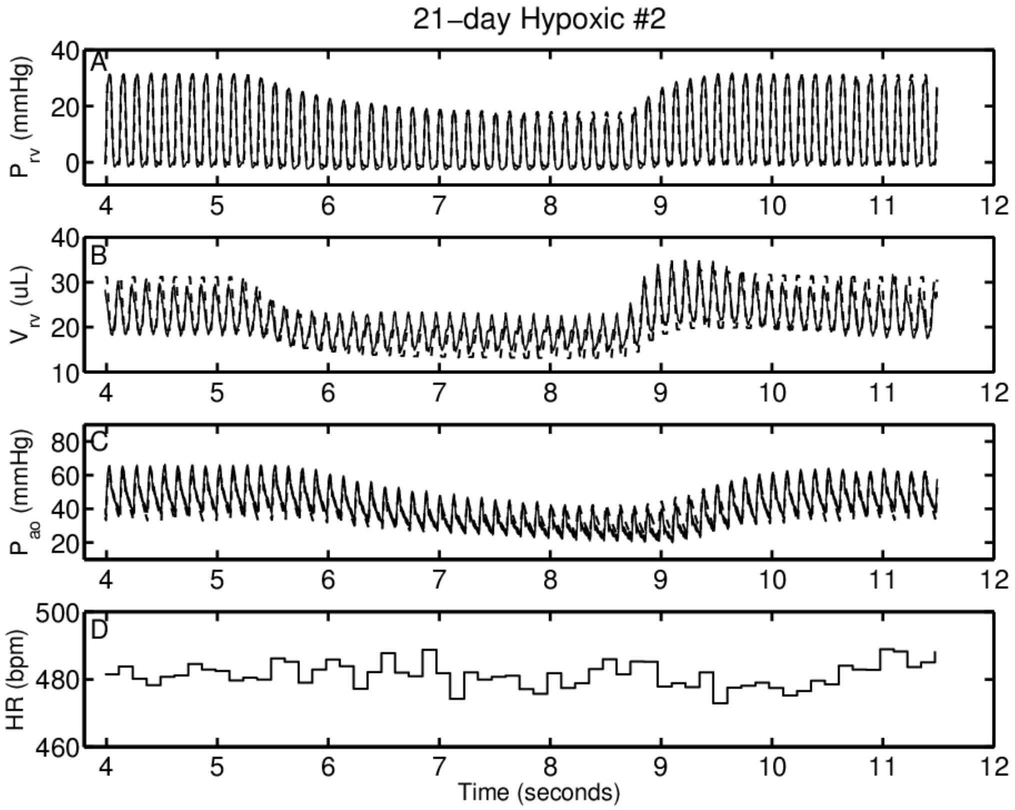

Figure S12:

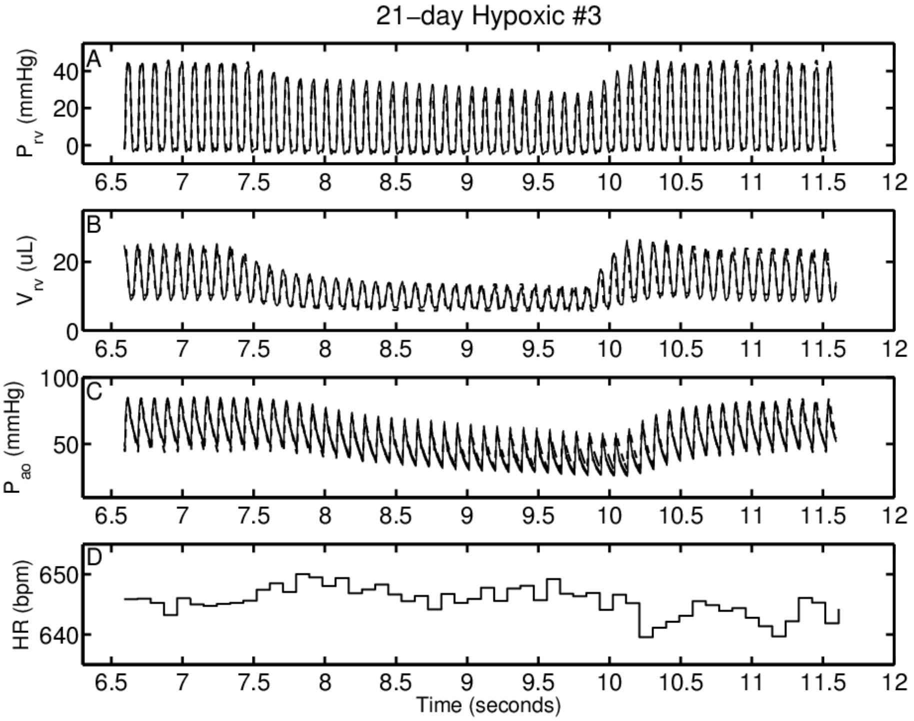

Figure S13:

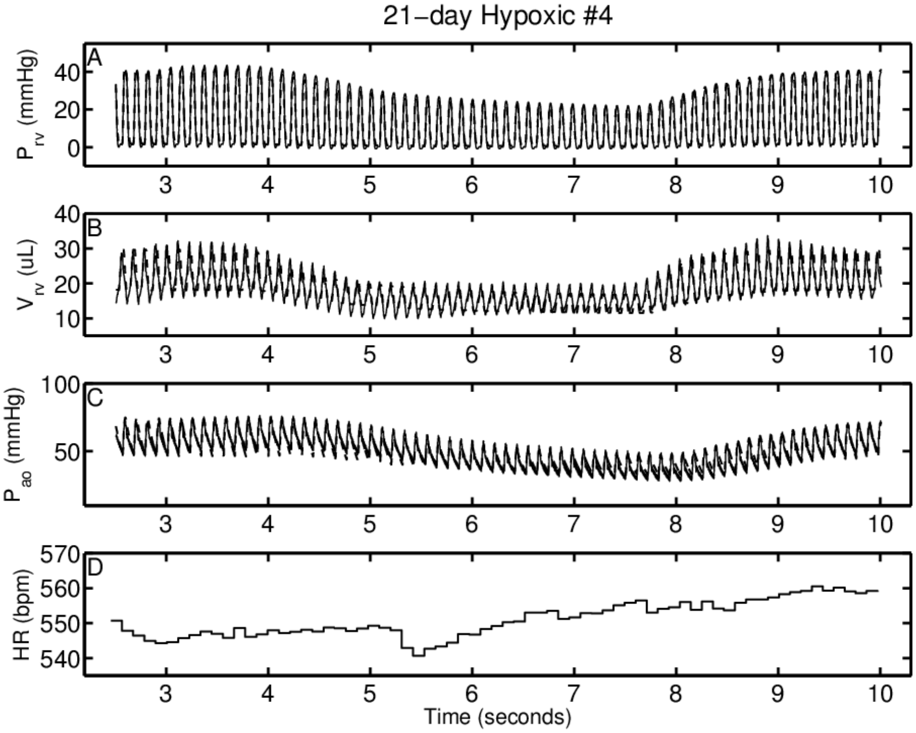

Figure S14:

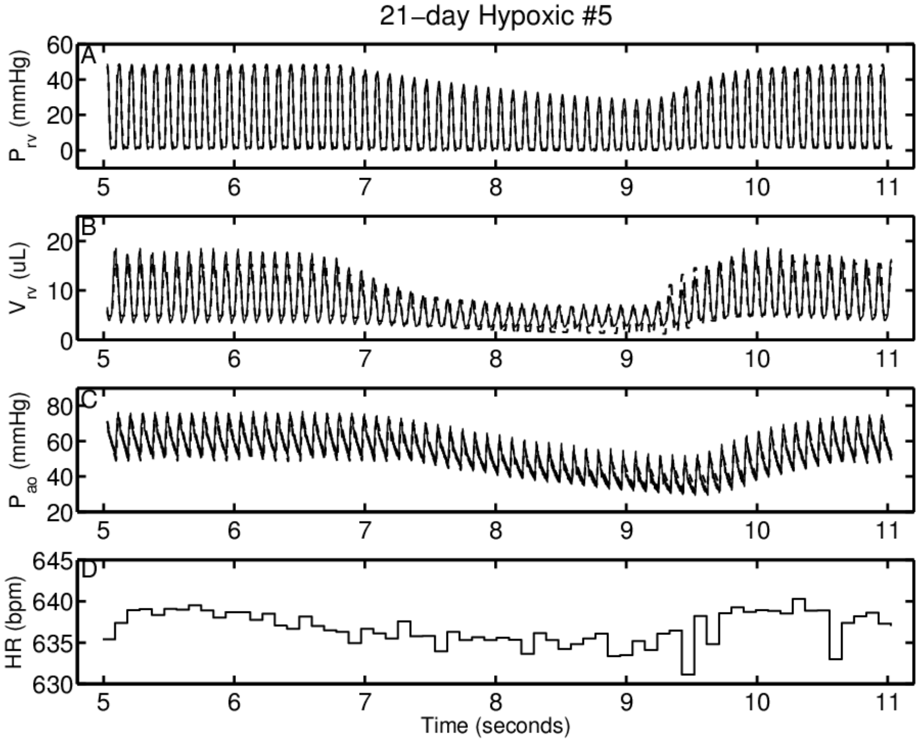

Figure S15:

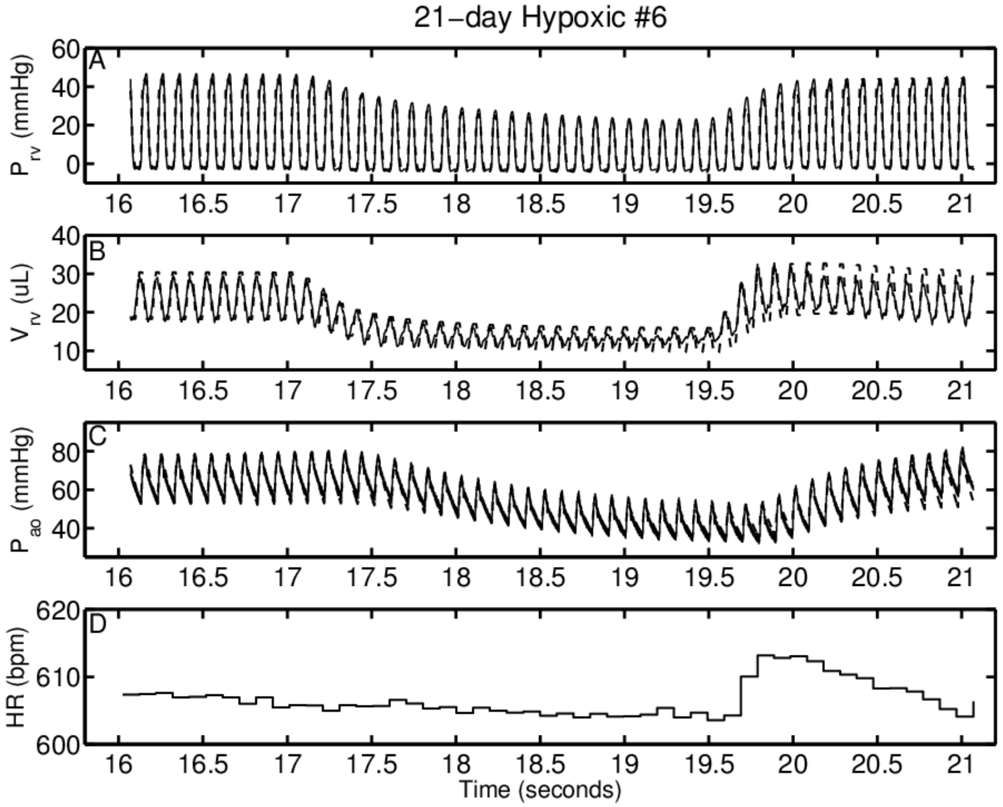

Figure S16:

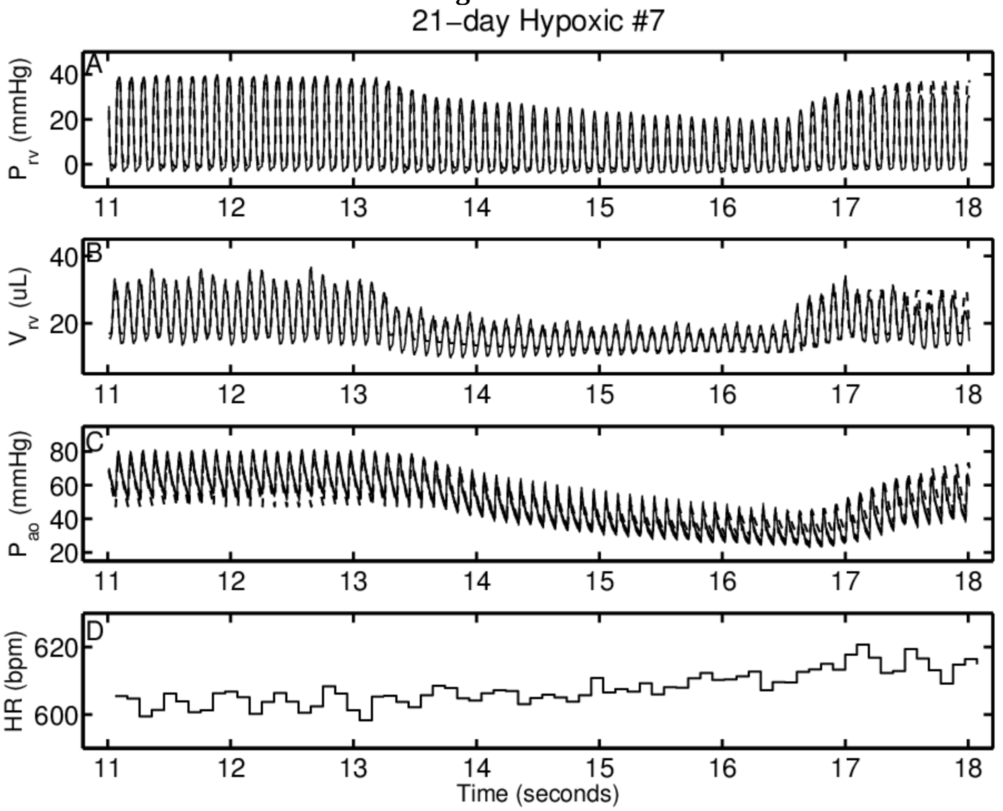

Figure S17:

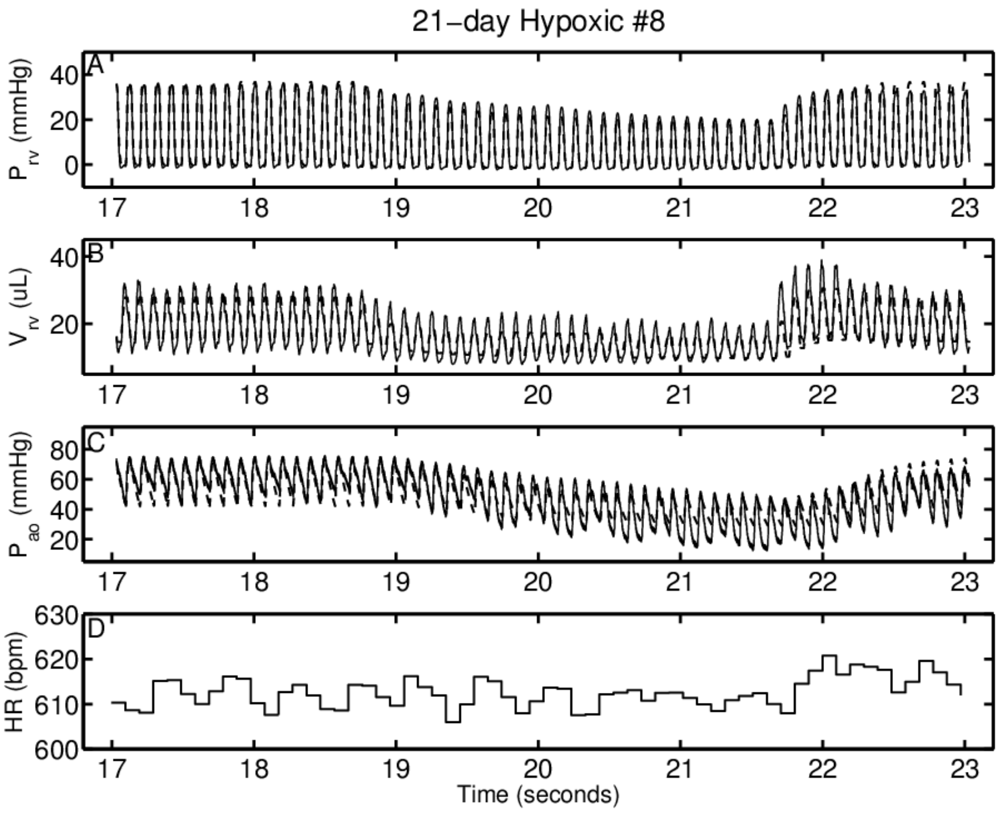

Figure S18:

e. <u>Experimental Data v/s Model Simulations of 28-day mice</u>

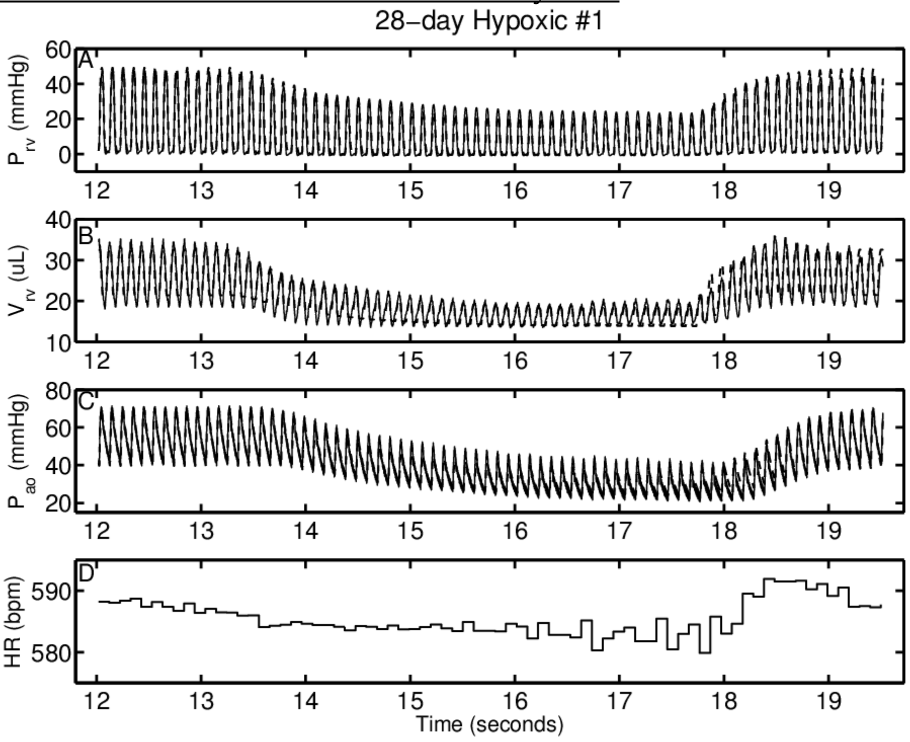

Figure S19:

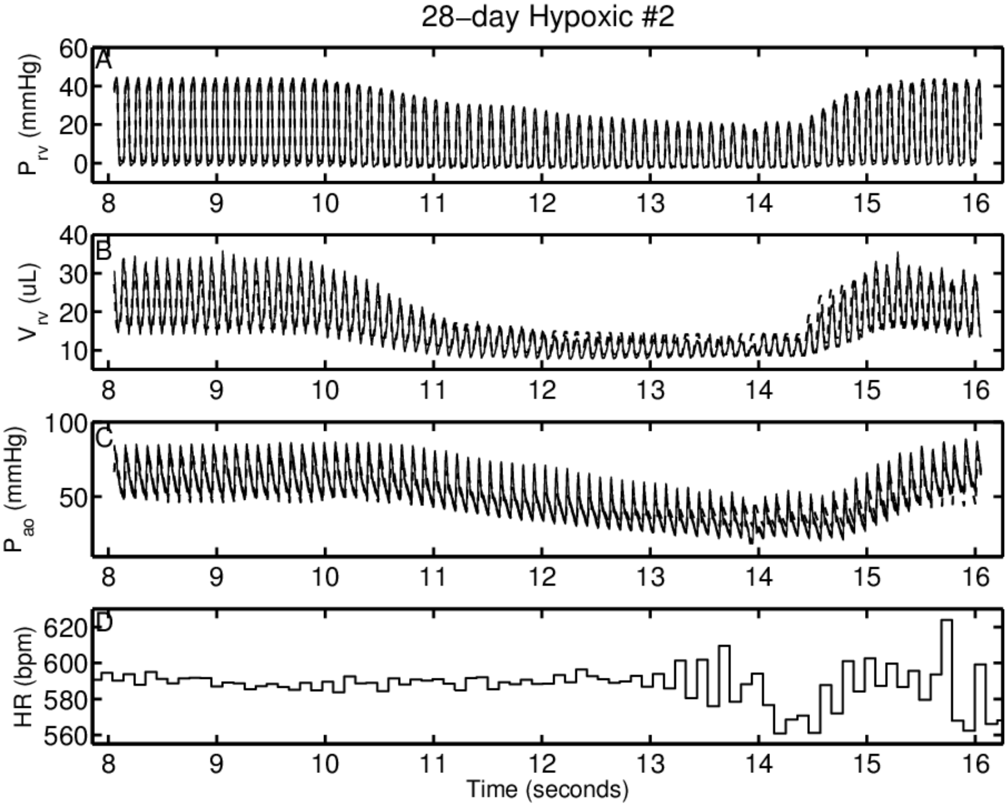

Figure S20:

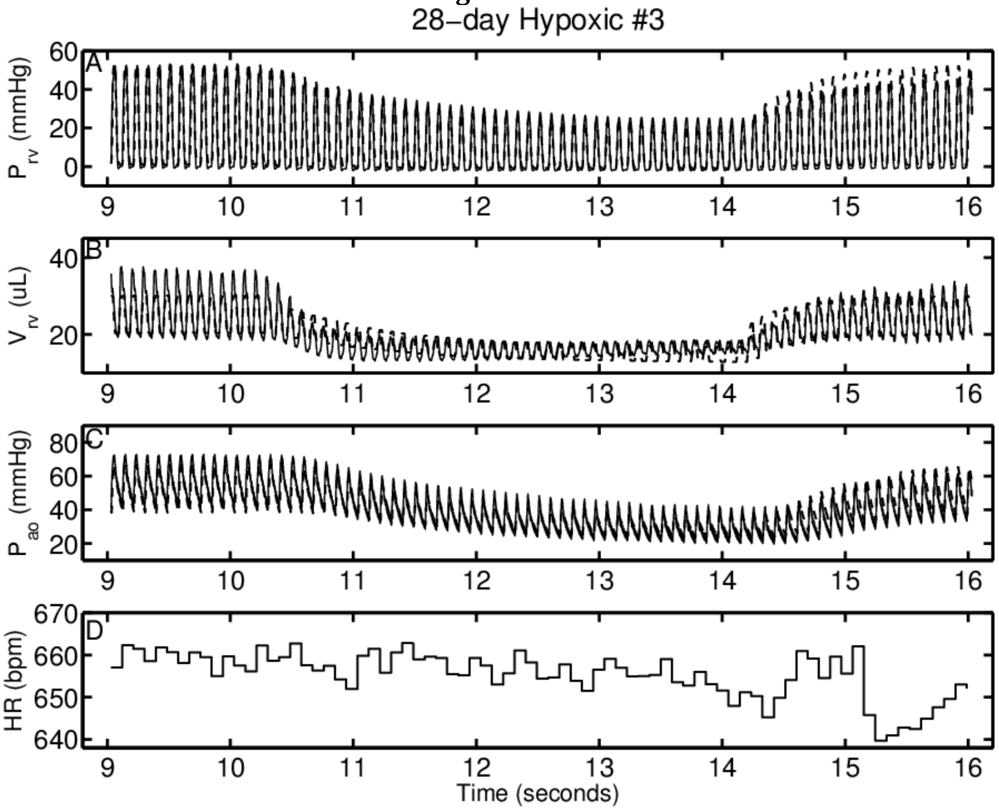

Figure S21:

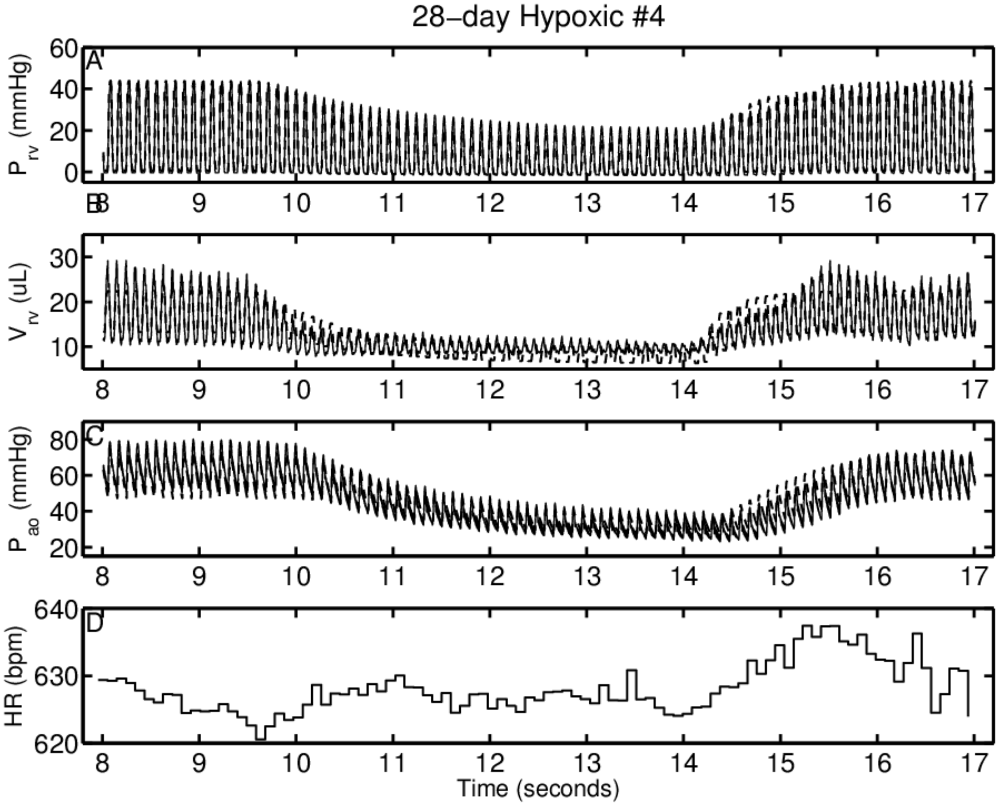

Figure S22:

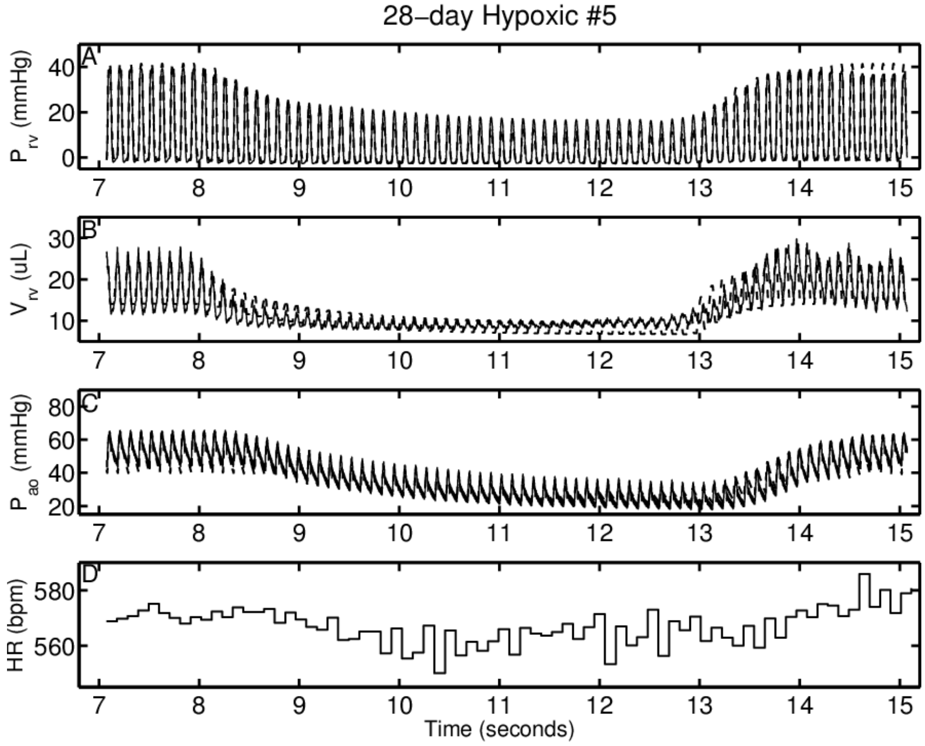

Figure S23:

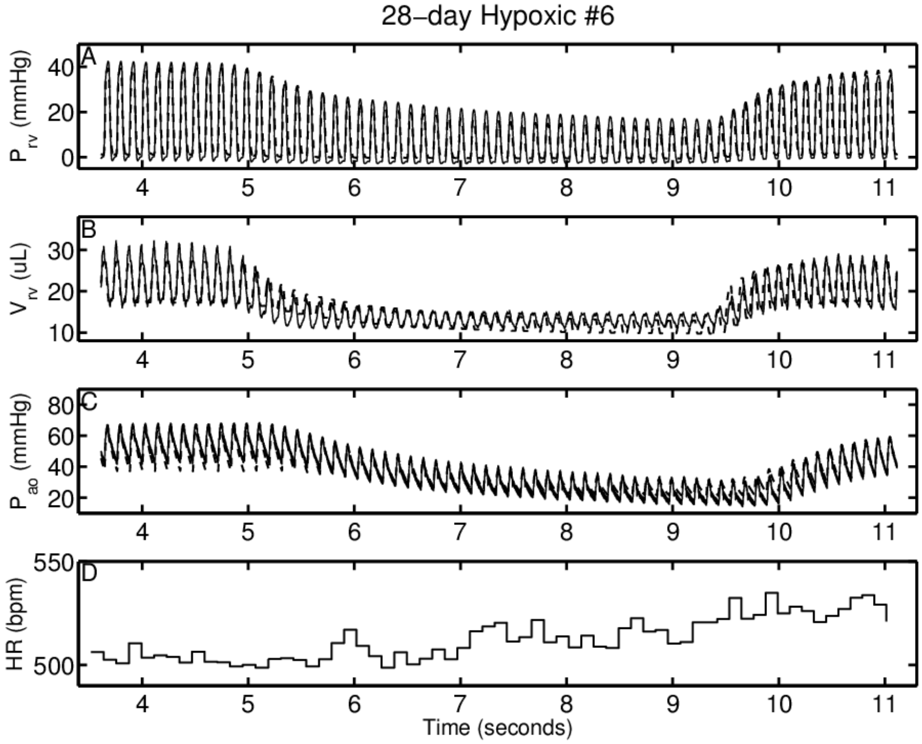

Figure S24:

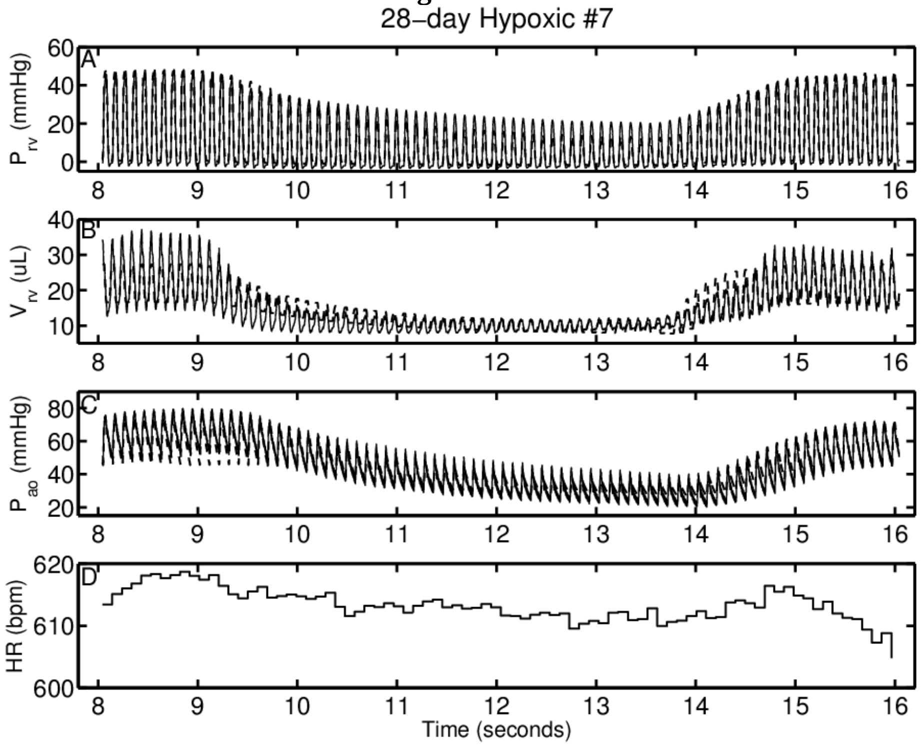

Figure S25:

Section 3: List of all parameters estimated/predicted

Parameter	0-day (n=4)	14-day (n=6)	21-day (n=8)	28-day (n=7)	Unit
**TriSeg Parameters**					
$A_{m,ref}^{LW}$ *	0.59 ± 0.07	0.59 ± 0.09	0.60 ± 0.06	0.61 ± 0.08	cm^2
$A_{m,ref}^{SW}$ *	0.10 ± 0.01	0.12 ± 0.02	0.11 ± 0.01	0.11 ± 0.01	cm^2
$A_{m,ref}^{RW}$ *	0.50 ± 0.01	0.57 ± 0.04†	0.59 ± 0.05†	0.60 ± 0.03†	cm^2
v_{max} *	12.36 ± 0.8	14.43 ± 4.2	14.1 ± 3.1	15.2 ± 3.8	μm/s
τ_D ‡	53.6 ± 2.9	53.5 ± 3.7	53.5 ± 3.1	55.8 ± 2.3	ms
τ_R ‡	76.6 ± 2.4	76.4 ± 3.0	75.2 ± 5.3	74.0 ± 1.0	ms
τ_{SC} ‡	437.1 ± 10.9	442.2 ± 19.3	439.7 ± 25.0	423.6 ± 14.9	ms
**Circulatory Parameters**					
R_{MT} ‡	3.56 ± 0.02	3.54 ± 0.10	3.51 ± 0.04	3.55 ± 0.06	mmHg·s/mL
R_{AV} ‡	0.013 ± 0.0001	0.013 ± 0.0003	0.013 ± 0.0001	0.013 ± 0.0002	mmHg·s/mL
R_{TC} ‡	0.019 ± 0.0001	0.019 ± 0.0005	0.019 ± 0.0001	0.019 ± 0.0003	mmHg·s/mL
R_{PV} ‡	12.024 ± 0.12	12.10 ± 0.4	12.132 ± 0.13	12.2 ± 0.25	mmHg·s/mL
R_{PVB} *	154.32 ± 11.1	286.52 ± 57.19†	265.5 ± 43.4†	356.6 ± 37.6†	mmHg·s/mL
R_{ABC} *	1442.6 ± 86.2	1946.0 ± 394.3	1769.2 ± 271.1	1883.3 ± 453.2	mmHg·s/mL
$R_{PBC}^{\dagger}$ *	569.6 ± 32.9	804.3 ± 121.9†	644.3 ± 63.5	741 ± 61.8†	mmHg·s/mL
Vd_{VC}*	0.45 ± 0.04	0.40 ± 0.072	0.43 ± 0.03	0.49 ± 0.05	mL
Vd_{IVC}‡	1.04 ± 0.05	1.0 ± 0.023	1.01 ± 0.05	1.01 ± 0.035	mL
Vd_{PA}*	0.16 ± 0.01	0.122 ± 0.028	0.121 ± 0.031	0.048 ± 0.03†	mL
Vd_{PU}‡	0.42 ± 0.03	0.42 ± 0.016	0.42 ± 0.02	0.43 ± 0.03	mL
Vd_{AO}‡	0.027 ± 0.001	0.027 ± 0.001	0.028 ± 0.001	0.027 ± 0.0006	mL
E_{VC}*	103.8 ± 6.6	103.8 ± 25.3	123.0 ± 45.5	69.8 ± 16.2	mmHg/mL
E_{IVC}‡	80.02 ± 4.3	83.0 ± 2.1	80.43 ± 3.04	77.7 ± 2.9	mmHg/mL
E_{PA}*	437.78 ± 26.6	560.3± 101.7	622.5 ± 172.1	938.6 ± 161.6†	mmHg/mL
E_{PU}‡	188.5 ± 10.2	193.9 ± 5.3	193.64 ± 8.46	185.2 ± 11.8	mmHg/mL
E_{AO}‡	3634.3 ± 91.7	3504.2 ± 87.5	3505 ± 448.0	3685 ± 97.7	mmHg/mL
**Predicted IVC Flow and estimated VCO %**					
pFLOW	71.6 ± 0.02	70.5 ± 0.02	73.1 ± 0.02	71.1 ± 0.05	-
*pVCO**	88.6 ± 1.4	81.21 ± 0.07	80.8 ± 0.04†	89.6 ± 0.03	-

† Indicates rejection of null-hypothesis at 5% significance level using two-tailed student's t-test. Note that statistical test was performed for all the parameters for differences with the control group.
‡ Globally fixed parameters.
* Individually estimated parameters.

Section 4: Contribution of R_{PVB} and E_{PA} towards RV Afterload and Preload

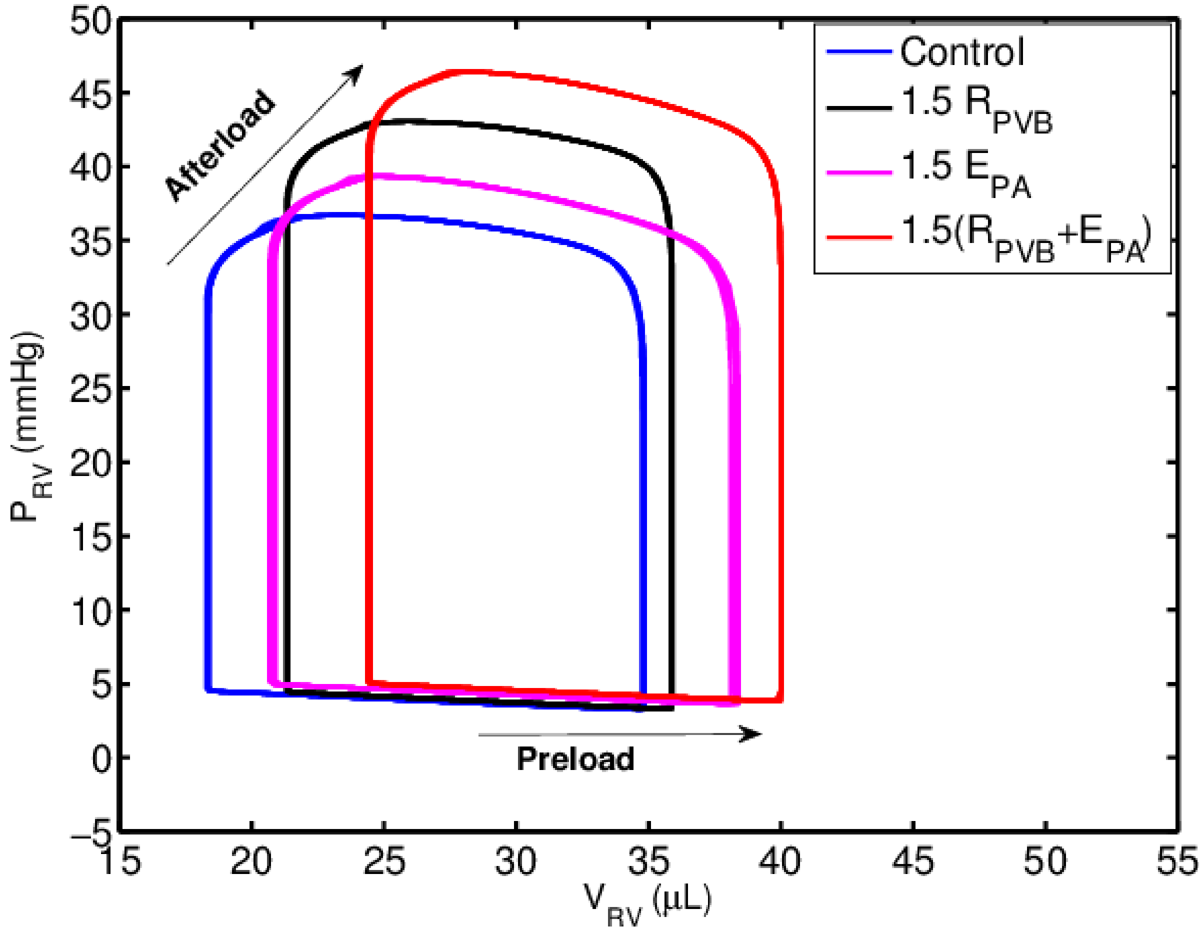

Figure S26: To delineate the individual contributions of pulmonary vascular resistance (R_{PVB}) and pulmonary artery elastance (E_{PA}), we performed model simulations with either R_{PVB} or E_{PA} or both set to 1.5 times their control values. Model simulations reveal that increased R_{PVB} and E_{PA} individually contribute more towards increased afterload and preload, respectively. However, the increases in afterload and preload are more prominent when R_{PVB} and E_{PA} are increased together.

References:

De Tombe, P.P., and Ter Keurs, H.E. (1990). Force and velocity of sarcomere shortening in trabeculae from rat heart. Effects of temperature. *Circ Res* 66, 1239-1254.

Hill, A.V. (1938). The Heat of Shortening and the Dynamic Constants of Muscle. *Proc R Soc Lond B* 126, 60.

Lumens, J., Delhaas, T., Kirn, B., and Arts, T. (2009). Three-wall segment (TriSeg) model describing mechanics and hemodynamics of ventricular interaction. *Ann Biomed Eng* 37, 2234-2255.

Ter Keurs, H.E., Rijnsburger, W.H., Van Heuningen, R., and Nagelsmit, M.J. (1980). Tension development and sarcomere length in rat cardiac trabeculae. Evidence of length-dependent activation. *Circ Res* 46, 703-714.
